# Narrative Review on Health-EDRM Primary Prevention Measures for Vector-Borne Diseases

**DOI:** 10.3390/ijerph17165981

**Published:** 2020-08-18

**Authors:** Emily Ying Yang Chan, Tiffany Sze Tung Sham, Tayyab Salim Shahzada, Caroline Dubois, Zhe Huang, Sida Liu, Kevin K.C. Hung, Shelly L.A. Tse, Kin On Kwok, Pui-Hong Chung, Ryoma Kayano, Rajib Shaw

**Affiliations:** 1 Collaborating Centre for Oxford University and CUHK for Disaster and Medical Humanitarian Response (CCOUC), The Chinese University of Hong Kong, Hong Kong SAR, China; huangzhe@cuhk.edu.hk (Z.H.); sida.liu@gxfoundation.hk (S.L.); kevin.hung@cuhk.edu.hk (K.K.C.H.); 2 Nuffield Department of Medicine, University of Oxford, Oxford OX37BN, UK; 3 JC School of Public Health and Primary Care, Faculty of Medicine, The Chinese University of Hong Kong, Hong Kong SAR, China; tiffany.sham@link.cuhk.edu.hk (T.S.T.S.); tayyabshahzada@link.cuhk.edu.hk (T.S.S.); shelly@cuhk.edu.hk (S.L.A.T.); kkokwok@cuhk.edu.hk (K.O.K.); chungpuihong@cuhk.edu.hk (P.-H.C.); 4 GX Foundation, Hong Kong SAR, China; caroline.dubois@gxfoundation.hk; 5 Accident & Emergency Medicine Academic Unit, The Chinese University of Hong Kong, Prince of Wales Hospital, Hong Kong SAR, China; 6 World Health Organization Centre for Health Development, Kobe 651-0073, Japan; kayanor@who.int; 7 Graduate School of Media and Governance, Keio University, Fujisawa 252-0882, Japan; shaw@sfc.keio.ac.jp

**Keywords:** health-EDRM, primary prevention, vector-borne disease, biological hazards, climate change, narrative review

## Abstract

Climate change is expanding the global at-risk population for vector-borne diseases (VBDs). The World Health Organization (WHO) health emergency and disaster risk management (health-EDRM) framework emphasises the importance of primary prevention of biological hazards and its value in protecting against VBDs. The framework encourages stakeholder coordination and information sharing, though there is still a need to reinforce prevention and recovery within disaster management. This keyword-search based narrative literature review searched databases PubMed, Google Scholar, Embase and Medline between January 2000 and May 2020, and identified 134 publications. In total, 10 health-EDRM primary prevention measures are summarised at three levels (personal, environmental and household). Enabling factor, limiting factors, co-benefits and strength of evidence were identified. Current studies on primary prevention measures for VBDs focus on health risk-reduction, with minimal evaluation of actual disease reduction. Although prevention against mosquito-borne diseases, notably malaria, has been well-studied, research on other vectors and VBDs remains limited. Other gaps included the limited evidence pertaining to prevention in resource-poor settings and the efficacy of alternatives, discrepancies amongst agencies’ recommendations, and limited studies on the impact of technological advancements and habitat change on VBD prevalence. Health-EDRM primary prevention measures for VBDs require high-priority research to facilitate multifaceted, multi-sectoral, coordinated responses that will enable effective risk mitigation.

## 1. Introduction

Vector-borne diseases (VBDs) are viral, parasitic and bacterial illnesses transmitted to humans through vectors such as mosquitoes, sand flies and ticks. Common VBDs affecting human health include malaria, yellow fever, dengue, Zika, chikungunya, Lyme disease, tick-borne encephalitis, leishmaniasis and African trypanosomiasis [1]. The complacency towards and reduced emphasis on vector control [2] and the redirection of health resources, together with population growth, urbanisation and globalization, have contributed to the increased frequency of VBD outbreaks in tropical areas of the world in the past decade [2]. With the impact of climate change on ecological and human living environment, the burden of VBDs has expanded from tropical and subtropical areas to temperate regions, placing 80% of the world’s population at risk [3]. This shift in the human vulnerability profile has been attributed to rising temperatures, which favour the migration and geographical expansion of disease vectors [4]. Furthermore, altered precipitation patterns favour larval breeding and have accelerated VBD spread [5]. Contact patterns between humans and pathogens, vectors or hosts may also be altered by climate change in an unpredictable manner [4]. Increased occurrences of natural hazards, such as floods and cyclones, pose a further risk of VBD outbreaks [4]. Geographical areas that were previously unaffected are now facing growing risks [6,7], but are often underequipped in disaster prevention, preparedness and response capacities.

The World Health Organization (WHO) estimates that VBDs currently account for over 17% of the global burden of infectious diseases [1]. As indicated in the Global Burden of Disease Study [8], VBDs have substantial disability weights [9] and can be detrimental to the socioeconomic development of communities. Malaria is a disease which accounts for more than 50% of total deaths caused by VBD [10], and high-risk countries have on average a gross domestic product per capita growth that is over five times lower than countries not affected by the disease [11]. The economic burden of VBDs stems from increased household expenditure on disease prevention and management, lost income from minimised productivity due to sickness or care for the ill [3], damages to crops and livestock by disease vectors [2], and other impacting factors. The United Nations Sustainable Development Goals (SDG) emphasise good health and well-being (SDG 3) [12]. Collaborative initiatives and investments prioritising prevention and treatment research by international bodies in recent decades, such as efforts by the Global Fund [13], have contributed to the alleviation of the global disease burden induced by VBDs [10].

The WHO health-emergency and disaster risk management (health-EDRM) framework was developed in 2018 as an integrated approach for the utilisation and management of resources in addressing current and emerging risks to public health, with the aim of promoting joint action and coherence in implementing other global strategies such as the International Health Regulations (2005), the Sendai Framework for Disaster Risk Reduction 2015–2030, the Paris Agreement on Climate Change, and the Sustainable Development Goals 2015–2030 [14]. Overall, the framework guides the structured analysis and management of health risks brought on by emergencies and disasters, focusing on risk mitigation through hazard and vulnerability reduction, preparedness, response, and recovery measures [14,15]. Health-EDRM emphasises the significance of community involvement to mitigating and counteracting the potential negative impacts of hazardous events such as VBD outbreaks, which are considered biological hazards [14].

The concept of prioritising health in disaster risk management policies was already recognised in the Sendai Framework for Disaster Risk Reduction 2015–2030 [16]. Health actors at all levels have engaged with each other and the WHO in the implementation and monitoring of disaster risk reduction. WHO offices at the regional level, and country governments, have incorporated disaster risk management policies in the health sector, which is an important step in contextualising actions for implementation [17]. The Sendai Framework has been crucial in highlighting health as a core dimension of disaster risk management, and has paved the way for the establishment of the WHO Health-EDRM Research Network, strengthening research and knowledge-sharing globally, allowing for the enhancement of evidence-based policies and practices [17]. There is a crucial need for multi-sectoral, coordinated approaches between the countries’ governments, health systems and other stakeholders, especially in the area of recording and reporting against the framework [17]. Additionally, systems need to reinforce the recognition of prevention and recovery within disaster management [17].

The health-EDRM framework outlines a hierarchisation of health risk prevention into primary, secondary and tertiary prevention [14,18]. Primary prevention mitigates against the onset of disease through health promotion targeted at behavioural modification and health risk reduction. Secondary prevention involves inhibiting disease progression through strategies such as screening and early detection. Tertiary prevention focuses on treatment and rehabilitation in order to minimise disabilities and complications [18,19]. Taking into consideration financial, clinical and infrastructural costs, primary prevention can effectively alleviate the burden of VBDs in a community, if necessary through measures that address a wide spectrum of VBDs, such as targeting diseases transmittable through multiple vectors [20] or focusing on vectors that are capable of transmitting multiple diseases [1]. Primary prevention measures often offer the most cost-effective outcomes and enhance health protection through increased community resilience against diseases where treatment is unavailable or access to healthcare is complicated. Secondary and tertiary prevention measures require significant human resources and health infrastructural support, and may therefore be costly, with higher programmatic risks, causing further economic stress on impacted communities.

There is a large amount of available evidence and research concerning clinical treatment approaches to some VBDs, such as Malaria. However, other VBDs, such as dengue, chikungunya, tick-borne encephalitis, Japanese encephalitis, yellow fever and leishmaniasis, lack standardised or straightforward treatments, and rely primarily on therapeutic interventions built on symptom management [21]. There are ongoing clinical trials in these areas, such as vaccine development for Zika and chikungunya, research into rapid malaria tests, as well as drug trials for chikungunya [22].

This narrative literature review examines published evidence on health-EDRM primary prevention measures for VBD risk mitigation, maps the contextual effectiveness or limitations of each preventive measure, and aims to identify areas of research that need be strengthened in order to develop effective strategies for VBD prevention. The strength of the available scientific evidence is evaluated for each of the prevention measures. Based on the health-EDRM framework, which emphasises the context-based determination of intervention efficacy, analysis of enabling and limiting factors is also included for each measure [14].

## 2. Materials and Methods

A keyword search-based narrative literature review was conducted using the databases PubMed, Google Scholar, Embase, Medline and ScienceDirect. The search was conducted in May 2020 and included English language-based international peer-reviewed articles, online reports, electronic books and press releases, as well as grey literature by institutions such as the WHO, the United Nations, the Global Fund, the United Nations Children’s Fund, the International Energy Agency, the World Bank, the United States Centres for Disease Control and Prevention, the U.S. Food and Drug Administration, and the Hong Kong Centre for Health Protection, published between January 2000 and May 2020. The snowballing search methodology was also applied. Specific keywords and phrases used can be found in Appendix A. The emergence, primary prevention, associated risk factors and management of VBDs were reviewed in order to generate 10 core primary prevention measures for discussion.

**Figure 1:**
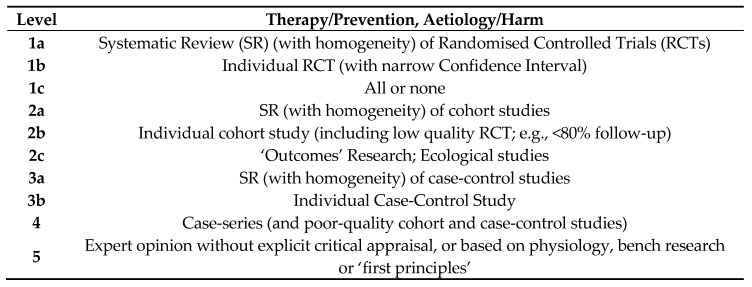
The Oxford Centre for Evidence-Based Medicine (OCEBM) 2009 Levels of Evidence (adapted from www.cebm.net) [23].

With reference to the Oxford Centre for Evidence-Based Medicine (OCEBM) 2009 Levels of Evidence (Figure 1) criteria, the identified papers were categorised into their respective levels according to strength of evidence based on the study design and methodology [23]. Reviewed literature that could not be categorised using the OCEBM Levels of Evidence was classified as ‘Others’, which includes, but is not limited to, news articles or releases, books, textbooks, position papers, guidelines, case reports and organisational reports.

## 3. Results

The search identified 134 relevant publications, all of which were included in the results analysis.

Using the identified research, 10 core bottom-up primary prevention measures were proposed and discussed based on the health-EDRM framework. Five personal protection practices (wear protective clothing when outdoors, avoid heading outdoors to vector-prone areas and during peak biting conditions, apply insect repellent, sleep under bed nets, receive prophylactic vaccinations and chemoprophylaxis), three environmental management practices (use insect-killing traps, manage stagnant water appropriately, manage waste appropriately), and two customary household practices (minimise household entry points, cover exposed foodstuffs) were included. Table 1 and Table 2 (personal), Table 3 (environmental) and Table 4 (customary household) highlight relevant health risk, desired behavioural change, potential co-benefits, enabling and limiting factors, alternatives, and strength of evidence available in published literature with regard to these primary prevention measures. Table 5 categorises all 134 reviewed publications according to the OCEBM Levels of Evidence [23]. Of note, a number of the reviewed articles report an assessment of more than one primary prevention measure. The review results indicate that approximately 60% of the studied literature relate to personal protection, 24% to environmental management, and merely 16% focus on customary household practices. Measures such as outdoor avoidance, sleeping under bed nets and receiving prophylactic vaccinations and chemoprophylaxis are amongst the most commonly reported studies. Details on the precise breakdown of each reviewed reference can be found in Table S1.

## 4. Discussion

VBDs are classified as biological hazards under the WHO health-EDRM framework [14] and their associated health risks should be managed according to the disaster management cycle (prevention, mitigation, preparedness, response and recovery), which encompasses both top-down and bottom-up interventions [157,158]. Top-down interventions require well-driven bottom-up initiatives to achieve effective primary prevention and to modify community health risk reduction-related measures [159]. Both the WHO health-EDRM framework [14] and the WHO global vector control response 2017–2030 framework [3] emphasise community engagement and mobilisation in enhancing protection against VBDs. The scientific effectiveness and feasibility of the community-level implementation of the 10 proposed primary prevention measures in this review can each be influenced by distinctive external factors, particularly with regards to access to financial or material resources.

Health promotion enables people to have more control over the improvement of their health outcomes, and is done through enhancing health literacy, encouraging behavioural change, and developing supportive policies [160]. There are numerous models which explore behavioural change as a result of education-based health promotion, one of which is the ‘knowledge, attitudes, practices model’, which prompts behavioural changes through knowledge enhancement [160]. In the case of vaccinations and chemoprophylaxis, it is critical for health interventions to enhance individual knowledge and awareness on why and how to receive prophylaxis as a primary prevention mechanism against VBDs, particularly in addressing misconceptions which underestimate the danger of VBDs [81]. Behaviour can be changed through addressing attitudes, such as misunderstandings [81], perception of social norms, cultural traditions and religious beliefs, for example in the case of ultra-orthodox Jewish communities who do not practice vaccination [81,82]. Finally, the behavioural change theory should consider how to promote practice. The viability and efficacy of the practice itself is favoured or limited by a variety of factors; policies will have to address barriers to accessing, and augmenting motivation in, the community [159].

The enabling and limiting factors that impact the effective uptake of primary prevention measures are closely interlinked. This review identified a number of determinants of success, including adequate resources, risk awareness, and well-coordinated supportive systems. A number of primary prevention measures rely on the availability and affordability of material resources, such as insect repellents, protective clothing, UV lamps, household building materials and bed nets (which additionally require space and equipment to set up [73]). Resource-deprived communities, which are at a higher risk of facing vulnerability, may lack the necessary material or financial resources. Materials must be accompanied by knowledge of their appropriate use. Inadequate information can lead to the improper maintenance of vector-prevention commodities, subsequently compromising their efficacy. For example, damaged bed nets with holes and improper bed net usage have been shown to lead to outcomes worse than no usage at all [64,65,66]. Some measures may also be affected by other health conditions, such as allergic reactions to insect repellent active ingredients [76], while others may be limited by cultural concerns, as demonstrated in the case of vaccination hesitancy in certain religious communities [81,82]. The feasibility of certain measures, such as the avoidance of outdoors, is dependent on an individual’s personal, professional and socioeconomic situation. Avoidance of going outdoors into vector-prone areas and during peak biting conditions can be impractical, such as in farming populations that need to spend long periods outdoors, and in tropical areas where the climate is ‘peak-biting’—hot and humid—all year long [50]. Similarly, there may be cases where access to a fully enclosed shelter or household improvements are not feasible, such as for those who are homeless or living in temporary shelters. Beyond resource access, proper education and personal circumstances, some primary prevention measures rely heavily on infrastructural and systemic support. Ensuring community access to vaccinations and chemoprophylaxis requires functioning health systems able to provide the necessary services, including an adequate supply of vaccines or medicine, trained health workers for administration and education, and an established clinic (fixed or mobile) from where the vaccine or drug can be distributed. Health system infrastructure is a critical enabling factor lacking in many rural or resource-poor contexts [84]. The environmental management of vectors also requires a robust and coordinated top-down waste management system [109,117], with multi-sectoral collaboration [161] between the health, environmental and civil engineering sectors, as well as other local and national-level authorities. Authorities should ensure the sufficiency of waste collection points such as waste bins [123], which can affect proper waste disposal, and the supply of electricity [118], which can affect the use of insect-killing traps, particularly in developing contexts [116]. Therefore, the success or failure of a community’s uptake of primary prevention measures is shaped by the availability of material resources and information, supportive health and civil infrastructure, policy formulation, geographical climate, individual or professional flexibilities, and social contexts. Nonetheless, it should always be noted that each measure offers its contribution towards VBD prevention, and the measures serve as an alternative to one another. When one measure cannot be carried out, the practice of other measures is not necessarily impeded.

In comparing the strength of evidence of the reviewed literature (Table 5, please see Table S1 for details), the largest proportion (45%) fell into Level 5 classification, which covers a wide range of study designs and methodologies, such as entomological studies, observational exploratory studies, experimental studies, modelling studies, qualitative studies, and expert opinions. 20% of the reviewed literature was categorised into ‘Others’, which includes but is not limited to news releases, reports by international organisations like the WHO, and textbooks. Level 4 publications, such as cross-sectional mixed method studies, behavioural surveys, household surveys, questionnaires, interventional studies and case series studies contributed a relatively large portion (17%), with many addressing the knowledge, perceptions, acceptance and opinions of populations with regards to VBD-prevention measures. Regarding individual primary prevention measures, evidence is most lacking at all levels with regard to the practices of covering exposed foodstuffs (4%) and proper waste management (6%). The literature relevant to sleeping under bed nets and minimising household entry points was significantly stronger in study design. There is published evidence on the risk reduction relating to wearing protective clothing and the management of stagnant water; however, while a multitude of studies emphasised the impact of primary prevention measures on VBD health risk reduction, a limited number of studies focused on the impact of the measure itself on disease prevention efficacy or outcome. For instance, many studies demonstrate the potential VBD-related health risks of exposed foodstuffs [136,137,138,139] and household entry points [140,141]; however, there are limited studies that demonstrate the effectiveness of covering food or household crack-repairing on disease incidence reduction within a community [156]. Similarly, for solid waste management, while evidence on the health risks [134,135] associated with improper solid waste accumulation is available, there is a lack of in-depth comparative studies between different waste management system models and their strengths and weaknesses.

The methodology used for this review is limited in that it does not include non-English-based literature, non-electronically-accessible literature, grey literature outside of those areas deliberately searched, any publications before 2000, or any publication not identified due to incompatibility with the keywords used for the literature search. Notably, publications documenting experiences from low-resource VBD-endemic settings that are not readily accessible via mainstream databases or online platforms may not have been included in this review.

Certain areas were found to be lacking in the updated evidence. On the efficacy of light-coloured clothing, while the WHO provides recommendations for protective wear against VBDs [21], the search generated no clear evidence, that had been updated within the past two decades, to support the rationale behind vector landing preferences on darker surfaces, and vice versa. Recommendations concerning the appropriate concentration of DEET in insect repellent are often inconsistent across international organisations and governments. More extensive research is needed to better establish the correlation between DEET concentration, repellent strength and duration of efficacy. In addition, while there are various observational studies on the correlation between modern technological advancements, such as air conditioning, and decreased disease vector bites [162,163,164,165], there is limited updated scientific evidence available on the precise impacts of such advancements on changes to vector habitat. Addressing these research gaps will facilitate better-grounded and more evidence-based institutional guidelines.

The best available evidence is always evolving, requiring the continuous updating of guidelines and recommendations. The ongoing research on VBD prophylactic strategies is very active, as well as that on the development of insecticide resistance regarding insecticide-treated bed nets [166,167] and insect repellents [168]. In light of the many different designs, parameters, sample sizes and investigation methods used, it is often difficult to evaluate and compare related studies, thus resulting in a lack of standardisation in guidelines. For instance, a variety of attraction and killing mechanisms, as well as door and window screen designs [141], are used in different studies to evaluate insect-killing trap and household modification efficacies. Efforts to achieve increased consistency in the methodology of published research are crucial to making comparative analyses between studies on different VBD-prevention commodities possible [169,170,171,172].

Three areas are particularly lacking in the published evidence. Firstly, there has been minimal research done on available alternatives to the proposed practices. Taking the case of insect repellents, numerous studies are available to prove the efficacy [59,60,61,85] and explore the potential safety concerns [86,87,88] of DEET. However, the strength of research supporting the repellence of natural alternatives like plant oils is variable [74]. For instance, limited and conflicting findings on citronella efficacy were identified [74,85], and potential health hazards, like dermatitis under high-concentration neem-oil use, are indicated, with less stringent safety testing conducted compared to DEET [74]. Secondly, limited research is available on other disease vectors such as sand flies and ticks. A bulk of the literature identified in this analysis focuses on mosquitoes—the discussions on common vector breeding grounds [52,106,107,108] and the efficacy of insect-killing traps seldom involve other disease vectors [128]. There is a need for research into effective methods to better understand the breeding habitat ecology of sand flies in immature stages, which will facilitate the development of targeted control strategies such as source reduction, which are not yet possible as sand fly larvae can be difficult to detect, in contrast to other vectors such as mosquitoes [173,174,175]. Similarly, in the case of insect-killing traps, only limited studies demonstrate their potential in targeting sand flies in addition to mosquitoes [129], and evidence on tick elimination by the traps is lacking entirely. Thirdly, research on the spectrum of VBDs is disproportionately distributed; studies are oftentimes skewed towards more prevalent VBDs, such as malaria. While consideration is given to other VBDs such as Zika or tick-borne encephalitis, this literature review occasionally extrapolates the primary prevention measures proposed for the more extensively-researched diseases so as to apply them to other VBDs as well—for example, the determination of the time of day with peak biting conditions was based on *Plasmodium*-infected (malaria) mosquitoes being active from dusk to dawn [29,30,31]. Further research on these three areas is necessary in order to develop comprehensive and informed guidelines or policies that can be implemented in varying contexts to mitigate against the risk and alleviate the disease burden of VBDs.

This review has identified major research gaps in the current published literature relating to health-EDRM primary prevention measures for VBDs (Table 6). Strengthening the available evidence in these areas will create a scientific basis on which governments, policy-makers and community stakeholders can develop effective, targeted and achievable strategies for protecting at-risk populations against VBDs. Aspects of the WHO health-EDRM framework can be applied to address these research gaps. Increasing capacities for information and knowledge management can support collection, analysis and dissemination across multiple sectors, allowing for the comparative evaluation of available evidence, as well as the development of consistent guidelines and recommendations [14]. This is particularly important for any research undertaken in resource-poor contexts, which will provide necessary evidence towards developing effective and targeted VBD prevention measures in such contexts. The framework highlights the need for more multifaceted and multisectoral approaches, the lessons of which will lead to the further development of evidence-based strategies [14].

All 10 primary prevention measures require sustainable, continuous implementation and maintenance in order to be truly effective in preventing VBDs. Primary prevention measures focusing on stagnant water, waste management and the covering of exposed foodstuffs offer the long-term co-benefit of mitigating risks arising from other biological hazards under the health-EDRM framework [14], such as water-borne and food-borne diseases [139]. Practising continuous primary prevention is particularly necessary as long as certain VBDs do not have standardised effective treatment options, and if vector-elimination is not feasible. Some preventive measures face more complex challenges in practise without adequate health or governance infrastructure. Others are more easily implemented, but are nonetheless reliant on materials such as insect repellents or bed nets, which can be an obstacle in resource-poor settings where the population is already facing vulnerability to impoverishment or disease. It is crucial for policymakers to ensure that systems are able to identify and assess needs, and provide the necessary support for the sustainable and fair distribution of resources. Empowering bottom-up initiatives requires well-coordinated top-down policies [83] that effectively disseminate resources and information, especially in resource-deprived, rural, or health-illiterate populations. A strong, accessible health system is key to providing materials and education to the at-risk population. Centralised, coordinated and well-regulated infrastructure, such as a uniform waste management system [176], can significantly enhance the efficacy of primary prevention practices.

Climate change and its associated consequences, such as changing weather patterns and increased disaster occurrences [18], have shifted the epidemiological patterns of VBDs, as well as the volume and spread of the at-risk population, thus affecting the development policies and strategies for mitigating the VBD burden on health systems. Rising temperatures and unpredictable precipitation patterns, for example, lengthen peak-biting periods and further complicate the capacity for outdoor avoidance, especially in tropical areas which are sultry throughout the year. The increased incidence of hydro-meteorological hazards such as floods and cyclones brings about more extreme rainfall, as well as increased humidity and water accumulation [18], and impact stagnant water management, thus possibly facilitating further larval habitat development for disease vectors [18]. Insect vectors cannot regulate their internal temperatures and are very sensitive to changes, which has caused them to invade new areas in order to adapt [177]. This puts previously unexposed populations at risk, who may lack protective immunity or the experience, resources or services necessary to mitigate the prevalence of disease [6]. The WHO health-EDRM framework stresses the importance of strengthening health systems, with an increased emphasis on climate change adaptation [14], to reducing health risks associated with hazardous events, including VBD outbreaks. It is important for governing bodies to consider the associated challenges of climate change during policy formulation, with the inclusion of climate change scenarios in disaster risk assessments [18]. Considering the limitation of the predicted impact of climate change on VBD transmission, governing bodies should enhance individual capacities and community resilience in cases of sudden VBD surges [178]. For instance, early warning systems should be in place to communicate the health risks associated with seasonal VBD outbreaks to vulnerable populations in advance [18]. As such, primary prevention measures that emphasise the broader aspects of environmental management, resource distribution and public education must not be overlooked. Public education, to encourage early symptom identification and subsequent health-seeking behaviours, can serve as a steppingstone in propagating secondary and tertiary VBD intervention amongst vulnerable populations.

In light of the growing burden of VBDs and emerging public health threats, a progressive primary prevention model is key to disaster risk reduction, as encompassed in the four priorities set out in the Sendai Framework for Disaster Risk Reduction (risk understanding, governance, preparedness and resilience) [16]. In terms of disaster risk understanding, a thorough examination of the enabling and limiting circumstances is required in at-risk populations, including local disease prevention capacity, specific VBD characteristics, and risk drivers such as climate change [16,18]. Disaster governance should be strengthened through stakeholder involvement and multi-sectorial collaboration, as well as through adopting a well-coordinated top-down approach to empowering bottom-up community initiatives in a sustainable manner. Resilience enhancement should be driven by global investments in innovation and research, for instance the development of better prophylactic strategies and better vector-prevention commodity designs for utilisation against VBDs. Finally, disaster preparedness can be reinforced through raised awareness, secured healthcare accessibility and health-seeking behaviour encouragement, so as to better equip vulnerable populations facing future VBD outbreaks.

## 5. Conclusions

This narrative study identified 10 health-EDRM primary prevention measures against VBDs. Resource availability, risk awareness and systemic support were identified as the core enabling factors for the success of these measures. Resources, health and civil infrastructure, policy formulation, geographical climate and socioeconomic factors were the core sources of limitations, which necessitate the need to consider alternatives. Evidence supporting the effectiveness of alternative preventive measures is lacking, in particular with regards to prevention in resource-poor settings. Similarly, evidence related to preventive measures focusses heavily on mosquitoes, whereas research on effective prevention against diseases transmitted by other vectors such as sand flies and ticks is lacking. At a global level, the necessity of VBD prevention increases with the growing impact of climate change and globalisation.

Health risks associated with VBDs will remain an ongoing biological hazard to communities, and thus sustainability of practice is crucial. As recommended by the WHO health-EDRM framework, in addition to the health sector, the successful adoption of primary prevention measures against VBDs requires a multi-faceted, multi-sectoral and coordinated response, encompassing sectors such as meteorology for hazard prediction, education for health awareness and promotion, and the environmental and civil engineering sectors for waste collection and water management.

In conclusion, this review has shown that evidence of the effectiveness and management of primary prevention practices is focused on a narrow spectrum of VBDs and vector types. In order to fill research gaps, the scope of VBD research should be broadened, and standardised protocols should be adopted so as to better prepare communities for disaster risk mitigation and to build the capacities of populations that are vulnerable with regards to health-EDRM practices.

## Figures and Tables

**Table 1:** Personal Protection Practices as Health Emergency and Disaster Risk Management (Health-EDRM) Primary Prevention Approaches against Vector-borne Diseases (VBDs) (Part 1).

Parametres	Wear Protective Clothing When Outdoors	Avoid Heading Outdoors to Vector-Prone Areas and During Peak Biting Conditions	
		Vector-Prone Areas	Peak Biting Conditions
**Risk**	Disease vectors have landing preferences for exposed skin over fabric. This is evident in studies on the Human Landing Catch (HLC) technique—HLC participants wear protective clothing to limit the area of exposed skin that attracts vectors [24]. There is often a greater risk of VBD transmission outdoors compared to indoors, as seen for malaria [25], chikungunya [26], and tick-borne disease transmission [27].	Specific locations such as secondary forests and rubber plantations are at a high risk of VBD transmission, such as dengue and Japanese encephalitis [28].	The time of the day and weather influence VBD exposure risk. Mosquitoes infected with *Plasmodium*, the parasite that causes malaria, are most active from dusk to dawn [29,30,31]. There are positive correlations between temperature and the number of dengue [32] and tick-borne encephalitis [33] transmission incidences, as well as between temperature, humidity, rainfall and the number of malaria transmission incidences [34].
**Behavioural Change**	Wear protective clothing, long-sleeve tops, and long trousers to minimise skin exposure and create a physical barrier against bites from vectors such as mosquitoes [31] and ticks [35]. Wear light-coloured clothing [21]. Wear loose-fitting and tightly-woven clothes to avoid vector bites through the clothing when it is pulled tight to the skin [36]. Tuck trousers into socks and boots to further reduce skin exposure. Seal clothing junctions with adhesive tape as an additional precaution under extreme infestation pressure [36].	Avoid vector-prone or VBD-endemic areas if possible [21,31,36,37].	Avoid or minimise outdoor activities during hot and humid seasons, unless necessary [37]. Avoid or minimise outdoor activities during specific periods of a day, such as from dusk to dawn in malaria-endemic areas if possible [21,31,36].
**Co-benefit(s)**	Protects skin from sun exposure and lowers risk of sunburn [38,39]. Protects skin from scratches and infections [39].	Reduces hazardous risks such as tiger [40] and bear [41] attacks in rubber plantations and secondary forests respectively.	Protects individuals from heat exhaustion and further progression to heat stroke under exposure to high temperatures [42,43]. Protects individuals from health risks such as increased cardiovascular disease mortality under exposure to high humidity [44]. Protects individuals from fall-related injuries, which are more prevalent during the rainy season [45].
**Enabling Factor(s)**	Availability and affordability of protective clothing [46]. Suitability of the weather—cool and dry weather is favourable where additional clothing is unlikely to cause discomfort.	Ability and flexibility to stay indoors for long periods without great discomfort; adequate household space is favourable. Ability to make informed decisions on specific local habitats and conditions to avoid; the risk variability of different environments and the non-exhaustive list of prone areas and peak biting conditions above should be noted.	
**Limiting Factor(s) and/or Alternative(s)**	Lack of protective clothing [46]. Presence of fabric holes in clothing: The holes serve as entry points for disease vectors to come into contact with skin. Holes may develop under the attack by fabric pests such as clothes moth larvae [47]. Unfavourable circumstances: In scorching areas and for labour-intensive occupations, heavy protective clothing may cause discomfort or impair human body heat exchange with the environment and cause heat stress [46,48].	Unfavourable circumstances: Staying indoors for long periods in poor, crowded living environments such as slums [49] may cause great discomfort.	
		Occupational limitations: Those such as farmers and rubber plantation workers do not have the flexibility to avoid prone areas.	Occupational limitations: Those with night shifts such as security guards and police officers do not have the flexibility to avoid heading outdoors at night.
			Unfavourable circumstances: For populations in areas which are typically sultry (hot and humid), such as the tropics [50], risk mitigation is more challenging.
**Strength of Evidence**	The effectiveness of wearing protective clothing as a physical barrier against vector bites is well-supported by evidence. While light-coloured clothing may enhance tick detection [37], it may also attract more ticks [51] and increase tick-borne disease risk. Findings on vector landing preferences on this matter are dated and inconsistent.	The positive correlation between larvae breeding and the extent of vegetation cover [52] is well-supported by evidence. The assertion that rubber latex collection cups in plantations are potential breeding sites for common vectors, especially during the rainy season [53], is well-researched.	The negative correlation between humidity and mosquito desiccation risk, as well as the positive correlations between temperature and larvae breeding, adult vector development and virus replication, are well-supported by evidence [54,55]. The relationship between temperature, humidity, rainfall, and vector transmission incidences is well-supported by evidence. Research on the relationship between time of the day and peak biting conditions is limited to malaria-transmitting mosquitoes. Minimal evidence is available on other VBDs and disease vector types such as ticks and sand flies.

**Table 2:** Personal Protection Practices as Health-EDRM Primary Prevention Approaches against VBDs (Part 2).

Parametre	Apply Insect Repellent	Sleep Under Bed Nets	Receive Prophylactic Vaccinations and Chemoprophylaxis
**Risk**	Vector landing rate is an indication of human biting rate of disease vectors [56], which is positively correlated with the risk of vector bites and subsequently VBD transmission.	Specific mosquito species tend to have higher biting rates at night [30]. An overwhelming majority of malaria vector bites occur when people are in bed [57].	The immune status of a population largely influences its sensitivity to diseases [58]. Immunologically-unprotected populations are particularly susceptible to infectious diseases [58].
**Behavioural Change**	Apply insect repellent on exposed surfaces (skin or clothing, but not on both simultaneously) in vector-prone areas, especially when outdoors [21,31,36,37]. Use repellent containing DEET, a common active ingredient that repels rather than kills mosquitoes [59,60] and ticks [61], thus minimising their chance of landing. Apply permethrin, another common active ingredient, to clothing. The chemical retains its effectiveness for up to six washings [62]. Use roll-on repellents as opposed to sprays [63]; the former minimises repellent dispersion to nearby foodstuffs and more effectively concentrates the repellent.	Sleep under bed nets in vector-prone areas [21,37]. Use bed nets, which offer an immediate physical barrier, to prevent disease vector entrance. Some bed nets are treated with insecticides, creating an additional chemical barrier to repel vectors. Ensure that bed net fabric is not in contact with the user [64] and no entry points are available for vectors [36]. Check the bed nets for holes, which may severely reduce their efficacy [64,65,66]. Select quality bed nets, which is essential to successfully prevent VBD transmission. Compared to conventionally-treated bed nets made by regularly dipping into insecticides [67], long-lasting insecticide-treated bed nets manufactured in factories have high efficacy and durability. Thus, the latter is recommended for long-term usage in vector-prone areas [67,68].	Receive the appropriate and up-to-date vaccine for those living in or travelling to vector-prone areas [21,37]. Vaccination is a form of active immunisation achieved through exposing an unimmunised individual to a pathogenic agent. The immune system is stimulated, and long-term immunity is achieved through triggering cell- or antibody-mediated immunity [69]. Receive the appropriate chemoprophylaxis recommended for those living in or travelling to vector-prone areas. Chemoprophylaxis is ‘the administration of a drug to prevent the development of a disease’ [70].
**Co-benefit(s)**	No other health co-benefits to note beyond its intended use.	Protects individuals from household pests such as rodents and cockroaches during sleep [64,71]. Prevents dust from landing on bed sheets and coverings [64]. Provides a sense of security through a closed sleeping environment, in particular for individuals living in open shelters.	Provides individuals with the opportunity to interact with health workers, access health services, and receive health education when visiting healthcare units for prophylaxis, in particular for remote, rural populations living in endemic areas.
**Enabling Factor(s)**	Availability and affordability of insect repellents. Proper education on the correct use of insect repellents.	Availability and affordability [72] of bed nets and related equipment for bed net hanging: Specific materials such as ropes and sticks [73] may be required to set up the bed nets. Availability of space to hang the bed net. Proper education on the correct use of bed nets [72].	Availability and affordability of vaccinations and chemoprophylaxis. Awareness and acceptance towards vaccinations and chemoprophylaxis. Accessibility of adequate and appropriate healthcare services.
**Limiting Factor(s) and/or Alternative(s)**	Lack of access to insect repellents: In resource-deprived areas, other potentially effective natural alternatives include eucalyptus-based repellents [74,75], neem [74] and citronella [74]. Potential health hazards: The active ingredients of insect repellents may cause allergy [76].	Lack of access to quality insecticide-treated bed nets: In resource-deprived areas, basic untreated bed nets, although not to the same extent, still offer significant protection from vectors as a physical barrier [77]. They may be constructed at home using mesh-like materials. Physical deterioration [64,65,66]: Damaged bed nets have significantly reduced efficacies. Proper maintenance of bed nets is important. Thermal discomfort [78,79]: Bed nets may attenuate airflow and cause discomfort to users, especially in hot and humid areas—this can be overcome with better designs. Inconvenience: The hanging [72,80] and washing [80] of bed nets may be considered troublesome. Complacency: People may underestimate the local severity and danger of VBDs [80], thus hold a complacent attitude towards the need for bed nets.	Vaccination hesitancy: ○ People may lack confidence in and be fearful towards vaccines (e.g., needle phobia), especially with the misunderstanding that vaccines pose a risk of infection [81]. ○ Vaccination may go against traditions and beliefs in specific social contexts or religions [81], such as in ultra-orthodox Jewish communities [82]. ○ People may underestimate the local severity and danger of VBDs, thus hold a complacent attitude towards the need for prophylaxis [81]. ○ People may have a preference for community-perceived alternatives to vaccines, such as alcohol, religious prayers and traditional remedies [81]. Lack of access [83] to prophylactic strategies: Inadequate vaccine supply, poor road terrain and inconvenient transport to immunisation centres, limited service delivery points, and insufficient health workers may hamper vaccination rates in developing countries [84]. Mobile immunisation campaigns may be preferred to reach poorly accessible areas [81]. Proper health educational interventions [81] and extensive vaccination programmes are crucial to enhance prophylaxis.
**Strength of Evidence**	Compared to other active ingredients in commercially-available insect repellents, DEET is well-supported to have a longer duration of efficacy [85]. Research on the safety of DEET has yielded conflicting findings. While some studies demonstrate potential harms such as the pro-angiogenic properties of DEET [86], others suggest that DEET imposes minimal to no evident health risks under proper usage [87], even when applied on vulnerable groups such as children and pregnant women [88]. Recommendations on the appropriate DEET concentration are inconsistent across international organisations and governments. Limited studies suggest that a higher DEET concentration indicates a longer duration of effectiveness, yet does not necessarily indicate higher insect-repelling ability [60,88]. More extensive research is necessary to establish a uniform DEET concentration recommendation across institutions. The strength of evidence available to support the efficacy of local natural alternatives is variable and may be conflicting, as in the case of citronella [74,85]. Local natural alternatives may also be subject to less stringent safety testing, as in the case of neem, which may cause dermatitis if used undiluted [74].	The strength of evidence available to support the efficacy of bed nets is strong. Bed nets demonstrate high potential for vector bite prevention in [89] vector-prone areas, such as for dengue and Japanese encephalitis [71,79]. The introduction of insecticide-treated bed nets has contributed to the substantial reduction in malaria transmission across sub-Saharan Africa [90]. Regarding bed net coverage, studies demonstrate that insecticide-treated bed net use in nearby compounds had a protective effect for child mortality and other health hazards in compounds lacking the bed nets, which suggests that high coverage of bed net use not only provides protection to individuals, but also has an area-wide effect on the mosquito population [91]. There is also evidence of the importance of widespread bed net coverage in the whole population for equitable community-wide benefits of protecting vulnerable target groups, such as young children and pregnant women, rather than merely exclusive bed net coverage amongst the vulnerable [92]. Bed net efficacy may be compromised under improper usage, such as incomplete net tucking and bed net sharing [64]. There is also evidence [64,93] of instances of mosquitoes biting through insecticide-treated bed nets, especially when users are in physical contact with the net fabric. There may further be a reduction in irritancy and toxicity of the insecticide-treated nets to mosquitoes after they feed on insecticide-treated bed net users [93], although further research is necessary to fully support this possibility.	The strength of evidence available to support the efficacy of VBD prophylaxis is variable. Some VBDs such as yellow fever [94,95], tick-borne encephalitis [96,97] and Japanese encephalitis [98,99] have highly efficacious vaccines that are well-supported by evidence. Some VBDs have limited prophylactic strategies available: ○ RTS,S, the only vaccine against malaria shown to be protective in young children, has been demonstrated to prevent 4 out of 10 cases of malaria in clinical trials [83]. Although RTS,S only offers partial protection and is a supplementary primary prevention strategy [83], pilot vaccination programmes have been or will be launched in three countries in sub-Saharan Africa [83,100]. ○ Malaria chemoprophylaxis, especially under long-term usage, may be associated with health risks, rare fatalities, adverse drug reactions and inadequacies [101,102], thus excluding chemoprophylaxis as a safe option for long-term travellers and populations in malaria-endemic locations and limiting its recommended use to short-term travellers [103]. Individualised strategies, such as sequential regimens with different medications for chemoprophylaxis, will have to be recommended instead [103]. Besides, vivax malaria relapses cannot be prevented with current first-line chemoprophylactic regimens [103]. ○ Dengvaxia, the only U.S. Food and Drug Administration (FDA)-approved vaccine against dengue fever [104], demonstrated poor efficacy [105], and may increase the risk of severe dengue symptoms in seronegative patients infected for the first time after vaccination, since it acts like a first dengue infection [105].

**Table 3:** Environmental Management Practices as Health-EDRM Primary Prevention Approaches against VBDs.

Parametre	Use Insect-Killing Traps	Manage Stagnant Water Appropriately	Manage Waste Appropriately
**Risk**	VBDs are transmitted to humans via living organisms such as mosquitoes, sand flies, and ticks [21].	Water bodies and still water are the most common mosquito larval habitats [52]; their prevalence increases the risk of disease transmission, as noted for VBDs such as Zika [106], chikungunya [107], and malaria [108]. Specific disaster occurrences may also increase VBD health risks. Under climate change, extreme weather events such as flooding and heavy rainfall may increase habitats for common vectors [20].	Accumulation and decomposition of solid waste attracts common houseflies, especially in areas with no centralised waste management systems and with open dumpsites [109]. Improper waste disposal augments the risk of VBD outbreaks.
**Behavioural Change**	Use insect-killing traps in areas with high-vector density [36]. Traps work by attracting and killing vectors. Select the appropriate trap for the context. Traps eliminate vectors by different mechanisms, such as emitting blue UV-light irradiation to increase reactive oxygen species production and damage DNA structures [110], and electrocuting insects on a high voltage grid. Dispose of dead insect bodies with care and proper hygiene such as thorough handwashing with soap and water after waste handling as they may carry VBDs and be hazardous.	Practice long-term habitual draining and elimination of stagnant water in containers inside and outside of households [21,31]. Take note of disaster-associated VBD health hazards in disaster-prone areas. Ensure that drinking water is stored in proper, sealed environments which are free of breeding potential.	Practice long-term habitual proper disposal of waste [21]. Practice specific waste management strategies such as the separation of organic and inorganic waste and the disposal of solid waste in open dumpsites away from water bodies, which are potential larval breeding grounds [111]. Microbial pathogens are prevalent in accumulated solid waste, and unprotected handling may result in infected wounds and sepsis [109,112]. Protect hands with gloves and/or use assistive tools such as clamps or tongs when handling waste. Wash hands thoroughly with clean water and soap after waste handling to minimise infection risk.
**Co-benefit(s)**	UV-light traps serve as an alternative light source due to their luminescent property.	Reduces the hazardous risk of slipping due to stagnant water on flooring [113]. Reduces the risk of mould development which has respiratory repercussions [114].	Encourages the separation of household waste which eases landfill burdens and reduces health hazards such as respiratory diseases and congenital abnormalities associated with proximity to landfills [115]. Reduces the arbitrary disposal of hazardous household waste [115]. Reduces surface water and groundwater pollution, air contamination, and greenhouse gas emissions (e.g., methane) from open waste dumping sites [116].
**Enabling Factor(s)**	Availability and affordability of insect-killing traps. Proper education on the correct use of insect-killing traps.	Availability of direct household water supply. Availability and affordability of tightly-sealed water containers.	A well-coordinated waste management system [117]. Availability and affordability of waste bags and bins.
**Limiting Factor(s) and/or Alternative(s)**	Lack of electricity: Insect-killing traps often rely on electricity to function. Taking the case of sub-Saharan Africa, nearly 600 million people have no access to electricity [118]. Passive non-electricity-requiring traps using fipronil-laced honey or toxic honey baits [119] to kill mosquitoes can potentially serve as alternatives. Lack of access to insect-killing traps: In resource-deprived areas, cheaper alternatives such as sticky paper traps with adhesive killing mechanisms can be used. However, their insect-trapping efficacy may be limited to closed environments such as greenhouses only [120].	Lack of water supply: It would be a challenge to avoid stagnant water accumulation in communities that lack direct household water supply—for these communities, it is common to store collected water from community standpipes and rivers [121]. Under such circumstances, tightly-sealed water containers are recommended for water storage. Lack of tightly-sealed water containers: For communities with only open plastic bottles or buckets available for water storage, larvicides can be added to the stagnant water. It is important to monitor the safety of the practice and educate people on the proper usage of larvicides [122].	Lack of a well-coordinated waste management system: Insufficient waste collection points and inadequate waste bins around the community, especially in developing countries [116] and resource-deprived areas, serve as barriers to proper waste disposal [123].
**Strength of Evidence**	A comparatively large amount of evidence on the working mechanisms and efficacy of insect-killing traps is available. A variety of attractants are used in insect-killing traps, such as blue UV-light [124], carbon dioxide [125], octenol [126] and heat [127], all of which are scientifically proven to draw insects. Studies have shown that different commercial insect-killing traps have varying efficacies in trapping and killing vectors such as the *Aedes* species, which can transmit chikungunya and Zika viruses [128]. Some traps can potentially target sand flies in addition to mosquitoes [129]. On the safety of different killing mechanisms, limited studies have demonstrated that the UV light in traps is non-hazardous to humans [130], whereas the electrocution of insects may potentially release bacteria and viruses [131]. Studies on whether or not pathogens remain in the infected dead insects’ bodies, and evidence-based guidelines on the proper disposal of dead insect bodies, are limited.	A comparatively large amount of evidence on the effectiveness of proper stagnant water management on VBD risk reduction is available. The aquatic characteristics of larval habitats are well-evidenced, and extensive research has been conducted regarding areas that are prone to stagnant water accumulation. Numerous studies demonstrate that household water containers, holes and furrows in discarded tyres [132], mud pots [116], and blocked drainage systems [109] are common larval breeding grounds. Case studies that evaluate the VBD outbreak risk associated with disaster occurrences that favour water accumulation are abundant. Taking the case of Djibouti, the country was suffering from pre-existing malaria and chikungunya outbreaks; studies reflect that heavy rain and floods in late 2019 further exacerbated the situation and exposed those affected to VBD risks [133].	A comparatively large amount of evidence on VBD prevalence in areas with improper solid waste accumulation is available. Items such as tyres, porcelain, plastic materials, and open coconut shells are commonly suggested to ‘provide breeding sites, burrows and food for vectors’ [134,135]. Such studies often link back to the favourability of larval breeding under stagnant water accumulation in waste materials [116,132]. There is also extensive research on how open dumping sites exacerbate VBD risks [116,134]. Evidence of the effectiveness of putting proper waste management into practice in communities and its relation to VBD risk reduction is minimal.

**Table 4:** Customary Household Practices as Health-EDRM Primary Prevention Approaches against VBDs.

Parametre	Minimise Household Entry Points		Cover Exposed Foodstuffs
	Wall Cracks	Door and Window Openings	
**Risk**	Household entry points such as wall cracks as well as open doors and windows provide opportunities for vector entrance, contributing to the risk of indoor infestation.		Common vectors such as flies are attracted to odours and chemicals released by exposed foodstuffs, such as the volatile fermentation products [136] of ripe fruits associated with the breeding of yeast in the fruit [137]. Disease vectors may contaminate exposed foodstuffs in open containers via direct contact or droppings, which contribute to health hazards such as a high incidence of diarrhoea in children under six [138]. If uncooked food with pathogens such as *Salmonella* and *E. Coli* are left uncovered, houseflies may serve as vectors and expose humans to the risk of food-borne pathogenic infections [139].
	A significant number of vectors may accumulate in the cracks if they remain unrepaired [140].	Entry points through open doors and windows have large surface areas and are more prone to the entrance of disease vectors [141].	
**Behavioural Change**	Household improvements to minimise entry points are effective in reducing infestation from vectors such as *Aedes aegypti*, which transmit the Zika and chikungunya viruses [142]. The risk of malaria transmission from the *Anopheles* mosquito is similarly reduced [143].		Practice the covering of exposed foodstuffs with food covers or nets to prevent food contamination by flies [111], especially in contexts without refrigerators.
	Repair cracks to seal potential vector entry points [21].	Install door and window screens and close windows in the early evening to reduce indoor disease vector density [21,36,144,145].	
**Co-benefit(s)**	Protects individuals from household pests such as rodents [146,147] and cockroaches [148].		Protects exposed foodstuffs from household pests such as rodents [149].
	Reduces water leakage [150], such as during heavy rainfall.	Enhances household safety, such as decreasing the risk of theft or burglary [151].	
**Enabling Factor(s)**	Availability and affordability of crack-repairing materials. Knowledge about crack-repairing, or accessibility to professional services.	Availability and affordability of door and window screens. Knowledge about door and window screen installation, or accessibility to professional services.	Availability and affordability of food covers.
**Limiting Factor(s) and/or Alternative(s)**	Contextual limitations: Household modifications do not apply to the homeless and the impoverished living in open, unstable shelters. Universal applicability: Household modification recommendations may not apply to all settings due to housing differences [152]. Professional requirement: Crack-repairing and door and window screen installation using modern methods often require professional tools and skills as well as long-term maintenance strategies.		Lack of access to quality food covers: In resource-deprived areas, clean pieces of cloth, lids, or any materials that can serve as physical barriers should be used as alternatives for covering exposed foodstuffs.
	Lack of access to modern crack-repairing materials: In resource-deprived areas, mud and lime mixtures may serve as alternatives, although they may be more costly in the long-term [153]. Less well-off populations that cannot afford modern building materials [154] may use other locally-available alternatives.	Lack of access to door and window screen installation services: The installation of door and window screens involves significant renovation work that is often costly and unaffordable for impoverished populations [155].	
**Strength of Evidence**	While there is available evidence on the effects of crack-repairing on VBD risk reduction, studies on the detailed evaluation of different crack-repairing methods remain limited. Materials such as cement, modern crack-fillers, and a mixture of mud and lime are scientifically proven to be efficacious in reducing indoor vector density. There are few studies on other more cost-effective alternatives for populations in resource-deprived areas. Mud is a locally-available alternative, but there are limited studies on whether crack-repairing with mud alone is potentially correlated with an increased risk of vector entrance [156].	A comparatively large amount of evidence on the efficacy of proper door and window screen installation, as well as the closing of windows, in reducing indoor vector density is available. Given that variations exist in screening designs, further research on their specific efficacies is necessary [141].	A comparatively large amount of evidence of the potential health risks associated with disease vectors if foodstuffs are exposed and not covered or stored well is available. Research on the efficacy of the use of food covers, and that of potential alternatives in resource-deprived areas, is limited.

**Table 5:** Overview of Health-EDRM Primary Prevention Approaches against VBDs in the Reviewed Articles, Categorised by the Oxford Centre for Evidence-Based Medicine (OCEBM) Levels of Evidence. (Please see Table S1 for details.).

Category	Intervention	Number of Reviewed Articles under Each Category in the OCEBM Levels of Evidence											
		1a	1b	1c	2a	2b	2c	3a	3b	4	5	Others *	Total
**Personal Protection Practices**	Wear Protective Clothing When Outdoors	0	2	0	0	0	0	0	1	4	4	3	14
	Avoid Heading Outdoors to Vector-Prone Areas and During Peak Biting Conditions	0	0	0	0	0	0	0	0	3	16	4	23
	Apply Insect Repellent	0	1	0	0	0	0	0	0	2	9	5	17
	Sleep Under Bed Nets	2	2	0	0	2	0	1	0	5	7	3	22
	Receive Prophylactic Vaccinations and Chemoprophylaxis	1	0	0	1	0	0	1	0	3	8	6	20
**Environmental Management Practices**	Use Insect-Killing Traps	0	1	0	0	0	0	0	0	0	8	5	14
	Manage Stagnant Water Appropriately	0	0	0	0	2	0	0	0	1	11	1	15
	Manage Waste Appropriately	0	0	0	1	1	0	0	0	1	4	2	9
**Customary Household Practices**	Minimise Household Entry Points	1	3	0	1	1	0	0	1	6	3	2	18
	Cover Exposed Foodstuffs	0	0	0	0	1	0	0	1	2	2	1	7
**Total**		4	9	0	3	7	0	2	3	27	72	32	159 **

* ‘Others’ includes but is not limited to news articles or releases, books, textbooks, position papers, guidelines, case reports and organisational reports.** Of the 134 publications reviewed, some included findings on more than one primary prevention measure, and are counted more than once in Table 5.

**Table 6:** Major Research Gaps in Current Published Literature Relating to Health-EDRM Primary Prevention Measures for VBDs.

	Research Gaps
1	Current studies on health-EDRM primary prevention measures for VBDs mostly focus on health risk reduction practices, yet efficacy evaluation on actual disease reduction is lacking.
2	Available literature is mostly classified as cross-sectional studies. Evidence on efficacy of the prevention measure based on randomised controlled studies or extensive cohort studies is limited.
3	Comparative evaluations for variations of certain primary prevention measures, such as efficacy of different insect-killing mechanisms or household modification materials, are limited.
4	Research outcomes are skewed towards certain vectors (e.g., mosquitoes). Research evidence on other vectors such as sand flies or ticks is limited.
5	Research outcomes are skewed towards certain VBDs (e.g., malaria). Research evidence on other VBDs such as Zika, chikungunya, or tick-borne encephalitis is limited.
6	Research and evidence on available alternatives to the proposed practices (e.g., using natural substitutes as opposed to chemical-based insect repellents) is limited.
7	Updated research on evidence relating technological advancements and the rapid change of ecological and human living environments to behavioural practices against VBDs is limited.
8	Consistency in recommendations from research papers, policies, and frontline international agencies (e.g., as in DEET concentration recommendations) is lacking.
9	Literature highlighting the effectiveness of multi-faceted, multi-sectoral and coordinated responses in enabling effective risk mitigation for population-level protection is lacking.

## Supplementary Materials

The following is available online at https://www.mdpi.com/1660-4601/17/16/5981/s1, Table S1: Relevant Intervention(s), Study Design, Relevant Key Finding(s) and/or Conclusion of Each Reviewed Article Referenced (*n* = 134).

## Appendix A. Keywords Used for Literature Search

‘bed nets’, ‘blue-light irradiation’, ‘bottom-up approach’, ‘breeding sites’, ‘carbon dioxide’, ‘cement’, ‘chemoprophylaxis’, ‘chikungunya’, ‘climate change’, ‘clothes moth larvae’, ‘clothes wear and tear’, ‘cockroaches’, ‘crack repair’, ‘dengue’, ‘diethyltoluamide (DEET) ’, ‘disease burden’, ‘door screening’, ‘doors and windows burglary’, ‘electricity access’, ‘fall injury water’, ‘floods’, ‘food decay’, ‘food fermentation’, ‘food mould and fungi’, ‘food-borne pathogens’, ‘forests’, ‘health hazards’, ‘health-EDRM’, ‘heat stroke’, ‘heat-seeking ability’, ‘heavy rain’, ‘household waste management’, ‘housing improvements’, ‘humidity’, ‘immunisation’, ‘infectious disease’, ‘insect repellents’, ‘insect traps’, ‘insecticide-treated nets’, ‘Japanese encephalitis’, ‘larval habitats’, ‘larvicides’, ‘lime’, ‘living environment’, ‘long clothing’, ‘long-lasting insecticide-treated nets’, ‘malaria’, ‘mosquito larvae’, ‘mosquito traps’, ‘mosquitoes’, ‘mould development water’, ‘mud’, ‘natural repellents’, ‘octenol’, ‘pesticide’, ‘primary prevention’, ‘protective behaviour’, ‘protective clothing’, ‘rodents’, ‘rubber plantations’, ‘sand flies’, ‘solid waste management’, ‘sticky traps’, ‘sunburns’, ‘temperature’, ‘tick-borne diseases’, ‘tick-borne encephalitis’, ‘ticks’, ‘top-down approach’, ‘tropical climates’, ‘ultraviolet irradiation’, ‘vaccination’, ‘vaccine complacency’, ‘vaccine hesitancy’, ‘VBDs’, ‘vector attraction’, ‘vector biting’, ‘vector contamination’, ‘vector exposure risk’, ‘vector human movement’, ‘vector landing preference’, ‘vector light clothing’, ‘vector net’, ‘vector traps’, ‘vectors’, ‘wall cracks’, ‘waste management’, ‘waste mismanagement’, ‘water storage’, ‘water supply’, ‘West Nile virus’, ‘window screening’, ‘yellow fever’, ‘Zika’.
