# Supplementary material for: Narrative Review on Health-EDRM Primary Prevention Measures for Vector-Borne Diseases
Source: Int J Environ Res Public Health. 2020 Aug 18;17(16):5981. doi: 10.3390/ijerph17165981 (PMC7459832; doi:10.3390/ijerph17165981)
Supplement: Supplementary file 1 [file ijerph-17-05981-s001.pdf]

Supplementary Materials

Table S1: Relevant Intervention(s), Study Design, Relevant Key Finding(s) and/or Conclusion of Each Reviewed Article Referenced (n=134)

Ref. No.	Title	Journal or Publication (Abbreviated)	Year of Publication	Relevant Intervention(s) (See Key 1)	OCEBM Levels of Evidence Based on Study Design (See Figure 1)	Relevant Key Finding(s) and/or Conclusion
21	A global brief on vector-borne diseases	WHO	2014	A, B, C, D, E, F, G, H, I, J	Others: A set of guidelines presenting a global brief on VBDs, which includes detailed profiles for different VBDs, prevention and control recommendations, and potential challenges.	• Details recommendations on VBD prevention and vector control including measures A, B, C, D, E, G, H, I, and J.
24	Considerations for the use of human participants in vector biology research: A tool for investigators and regulators	Vector-Borne Zoonotic Dis	2015	A	Level 5: A research methods tool to guide investigators, as well as scientific and ethical review committee members on vector biology research involving human participation.	• Recommends the wearing of protective clothing to cover parts of exposed skin for HLC participants and minimise risks of potentially infectious vector bites.
25	Higher risk of malaria transmission outdoors than indoors by *Nyssorhynchus darlingi* in riverine communities in the Peruvian Amazon	Parasites and Vectors	2019	B	Level 4: A cross-sectional study involving four communities to gather baseline information about bionomics of malaria vectors and transmission risk factors in a hyperendemic malaria area of Amazonian Peru.	• Suggests that malaria transmission risk is higher outdoors than indoors in the studied communities.
26	Risk Factors for Symptomatic and Asymptomatic Chikungunya Infection	Trans R Soc Trop Med Hyg.	2013	A	Level 4: A cross-sectional study on the risk factors for symptomatic and asymptomatic chikungunya infection.	• Concludes that being in close proximity to a garbage pile and spending at least eight hours per day outdoors were associated with increased symptomatic and asymptomatic chikungunya infection.<br>• Suggests that individuals aged greater than 58 and with a high level of formal education were less prone to symptomatic chikungunya infections.
27	Incident Tick-Borne Infections in a Cohort of North Carolina Outdoor Workers	Vector-Borne Zoonotic Dis.	2016	A	Level 1b: A randomised controlled trial to evaluate the effectiveness of long-lasting permethrin-impregnated clothing in reducing tick-borne disease infection risks amongst North Carolina outdoor workers.	• Concludes that subjects wearing permethrin-impregnated clothing had a lower risk of tick-borne disease infection, though not statistically significant.<br>• Suggests that outdoor workers in North Caroline at a high risk of tick-borne disease infections, with many of the cases remaining asymptomatic.

28	Risk of exposure to potential vector mosquitoes for rural workers in Northern Lao PDR	PLoS Negl Trop Dis	2017	B	Level 4: A cross-sectional behavioural survey and mosquito sampling to compare vector exposure risk in villages, secondary forests, or rubber plantations.	• Indicates that compared to staying in villages, entering secondary forests or rubber plantations present with a higher risk of dengue vector exposure.<br>• Encourages human behaviour understanding to identify risky behaviours and vector control expansion to include rubber plantations.
29	Dusk to dawn activity patterns of *Anopheline* mosquitoes in West Timor and Java, Indonesia	Southeast Asian J Trop Med Public Health	2011	B	Level 5: An insect study capturing adult mosquitoes to assess their activity patterns in different locations and time periods of a day.	• Indicates that a general pattern on the studied disease vectors show peak activities during night time from dusk to dawn.
30	Malaria transmission and vector behaviour in a forested malaria focus in central Vietnam and the implications for vector control	Malar J	2010	B	Level 5: A entomological survey on adult mosquitoes to assess malaria transmission rates and behaviour of disease vectors in different locations and time periods of a day.	• Suggests that malaria transmission risks are high throughout the entire night, from dusk to dawn.<br>• Recommends a combination of vector control measures such as long-lasting insecticidal hammocks for primary prevention.
31	Protective behaviour and West Nile virus risk	Emerg Infect Dis	2005	A, B, C, G	Level 4: A cross-sectional, household survey on the association between outdoor exposures, personal or source-reduction protective behaviour and West Nile Virus risk as well as the relevant risk factors.	• Indicates that increased time spent outside at dusk or dawn is a significant risk factor for infection.<br>• Indicates that personal behaviours including the lack of vector-prone area avoidance, outdoor exposure avoidance, protective clothing (long sleeves or long pants) when outdoors and mosquito repellent use when outdoors are associated with increased infection risks.<br>• Indicates that the practising of two or more personal protective behaviour traits was associated with significantly reduced infection risks.<br>• Indicates that source-reduction behaviours including stagnant water drainage and gutter checking and cleaning are associated with decreased infection risks.
32	The global distribution and burden of dengue	Nature	2013	B	Level 5: A modelling framework mapping global distribution of dengue risk, paired with dengue cohort studies and population surfaces to infer public health burden of dengue in 2010.	• Infers a strong association between high levels of precipitation, high temperature and elevated dengue risk.

33	Impact of air temperature variation on the ixodid ticks habitat and tick-borne encephalitis incidence in the Russian Arctic: the case of the Komi Republic	Int J Circumpolar Health	2017	B	Level 5: A study on the relationship between tick-borne encephalitis incidence and changes in different external factors.	• Indicates that the expansion of the range of *Ixodes persulcatus* contributes to the rise in tick-borne encephalitis incidence in recent years.<br>• Concludes with a positive correlation between high air temperature and tick-borne encephalitis incidence, with climate change being a driver of incidence rate growth.
34	The association between temperature, rainfall and humidity with common climate-sensitive infectious diseases in Bangladesh	PLoS One	2018	B	Level 5: An observational exploratory study on the association between climate-sensitive infectious disease occurrence and climate characteristics.	• Infers a strong association between high levels of precipitation, high temperature, high humidity and malaria incidence.
35	Effectiveness of Personal Protective Measures to Prevent Lyme Disease	Emerg Infect Dis	2008	A	Level 3b: A case-control study to evaluate the effectiveness of personal protective measures such as protective clothing on the prevention of Lyme disease.	• Suggests that protective clothing was 40% effective.<br>• Suggests that tick repellent usage directly on skin or on clothing (upon routine usage) was 20% effective.<br>• Concludes that the use of protective clothing and tick repellents are effective in the prevention of Lyme disease.
36	Global Collaboration for Development of Pesticides for Public Health (GCDPP) Repellents and Toxicants for Personal Protection	WHO	2000	A, B, C, D, F, I	Others: A position paper on personal protection practices against VBDs.	• Details recommendations on VBD prevention and vector control including measures A, B, C, D, F, and I.<br>• Indicates that loose-fitting and tightly woven clothing can prevent mosquito bites through the fabric when it is drawn tight to the skin.<br>• Indicates that trousers should be tucked into socks and boots; under extreme infestation pressure, adhesive tape can be used to seal clothing junctions.
37	Lyme disease: Current issues, implications and recommendations for tourism management	Tour Manag	2015	A, B, C, D, E	Level 5: A literature review on the risk factors of Lyme disease and corresponding tourism management implications.	• Indicates that wearing light-coloured clothing makes it easier to detect and remove ticks.<br>• Mentions the effectiveness of primary prevention measures such as wearing protective clothing, applying insect repellent, and sleeping under bed nets.
38	Hat, shade, long sleeves, or sunscreen? Rethinking US sun protection messages based on their relative effectiveness	Cancer Causes Control	2013	A	Level 4: A cross-sectional study on the association between sunburn and sunscreen use, shade-seeking, and protective-clothing-wearing.	• Concludes that long-sleeved clothing and avoiding the sun by seeking shade significantly lower the odds of multiple sunburns.

						• Suggests that shade-seeking and wearing of protective clothing may be more effective than sunscreen for protection.
39	Big Book of Self-Reliant Living: Advice And Information On Just About Everything You Need To Know To Live On Planet Earth	Rowman & Littlefield	2004	A	Others: A book on self-protection measures, specifically relating to wearing protective clothing.	• Indicates that protective clothing protects skin against infections caused by scratches and sunburns.
40	Sumatran tigers seen on plantation in Riau	The Jakarta Post	2019	B	Others: A news article on the presence of Sumatran tigers on rubber plantations.	• Indicates potential hazard of tiger attacks in rubber plantations.
41	Habitat selection of a large carnivore along human-wildlife boundaries in a highly modified landscape	PLoS One	2014	B	Level 5: A study on the behaviour of bears in different seasons, including their habitat selection preferences.	• Demonstrates that bears selected habitats in close proximity to areas such as abandoned forests, agricultural lands, and rivers.<br>• Indicates potential hazard of bear attacks in secondary forests.
42	Relationship between weather factors and heat stroke in Ningbo city	Chinese J Endem	2016	B	Level 5: A modelling study on the correlation between heat stroke incidences and weather factors.	• Concludes a positive relationship between high temperature and the risk of heat strokes.
43	Heat exhaustion	Handbook of Clinical Neurology	2018	B	Others: A set of guidelines on heat exhaustion.	• Suggests that heat exhaustion may progress to heat stroke.<br>• Suggests a positive correlation between prolonged hot environment exposure and heat exhaustion.
44	Humidity may modify the relationship between temperature and cardiovascular mortality in Zhejiang province, China	Int J Environ Res Public Health	2017	B	Level 5: A modelling study on the correlation between cardiovascular mortality and weather factors.	• Concludes a positive relationship between high humidity, low temperature and cardiovascular mortality.
45	Effect of weather and time on trauma events determined using emergency medical service registry data	Injury	2015	B	Level 5: A modelling study on the correlation between trauma admissions and weather or temporal factors.	• Concludes a positive relationship between increased magnitude of precipitation and fall-related injury occurrences.
46	Acceptability of insecticide-treated clothing for malaria prevention among migrant rubber tappers in Myanmar: a cluster-randomized non-inferiority crossover trial	Malar J	2017	A	Level 1b: A cluster-randomised non-inferiority crossover trial to determine the acceptability of insecticide-treated clothing versus identical, untreated clothing among migrant rubber tappers through structured questionnaires, focus group discussions and in-depth interviews.	• Concludes that there is a high level of acceptability towards insecticide-treated clothing as a strategy for personal protection amongst rubber tappers.<br>• Suggests that 'too hot' and 'not having long clothes available' were significant reasons for not wearing long clothes for work.<br>• Indicates reservations about cost when purchasing protective clothing and encourages

						future research into cost-effectiveness and potential relevant financing mechanisms.
47	Susceptibility of environmentally friendly sheep wool insulation panels to the common clothes moth *Tineola bisselliella* in laboratory assays	Insects	2019	A	Level 5: A laboratory study on the susceptibility of sheep wool panels to attack by common clothes moths.	• Indicates that clothes moth larvae have the potential to attack clothing fibres and create holes.
48	Occupational heat stress assessment and protective strategies in the context of climate change	Int J Biometeorol	2018	A	Level 5: A review on occupational heat stress under different protective strategies in the context of climate change.	• Indicates that protective clothing has the properties of thermal insulation and evaporative resistance, thus may impair human body heat exchange in hot environments.<br>• Indicates that high physical work intensity correlates with internal metabolic heat production in the body.
49	Slum Health: From Understanding to Action	PLoS Med	2007	B	Level 5: A review on the general condition of slums as well as potential challenges faced and actions to be taken by populations living in such areas.	• Indicates that overcrowding is a common phenomenon in slums with high occupancy rates and crowded living environments.
50	Tropical Weather	Nat Educ Knowl	2012	B	Level 5: Expert opinion on the characteristics of tropical weather.	• Indicates persistent hot and humid climates in the tropics.
51	Detecting ticks on light versus dark clothing	Scand J Infect Dis	2005	A	Level 4: An observational study to compare the attractive effect of light and dark clothing on tick landing.	• Suggests that light-coloured clothing attracts more ticks than dark-coloured clothing.
52	Characterization of Mosquito Breeding Sites in and in the Vicinity of Tigray Microdams	Ethiop J Health Sci	2011	B, G	Level 5: An investigation on mosquito larval breeding habitat characteristics and preferences.	• Indicates larval breeding preferences in areas with vegetation cover, rainfall, water bodies and fauna.
53	Species diversity of mosquito breeding in rubber plantations of Kerala, India	J Am Mosq Control Assoc	2012	B	Level 5: An investigation on the mosquito larval breeding preferences in rubber plantations in Kerala.	• Suggests that multiple species of mosquitoes favour breeding in rainwater-filled cups used for collecting rubber latex in plantations.
54	Effect of climate and weather on the transmission of Ross River and Murray Valley encephalitis viruses	Microbiol Aust	2000	B	Level 5: A review on the effects of climate and weather on Ross River and Murray Valley encephalitis virus transmission via disease vectors.	• Indicates that humidity is crucial to mosquito survival and longevity.<br>• Indicates that low humidity increases the risk of desiccation in mosquitoes.<br>• Indicates that higher temperature facilitates higher rate of larval breeding, especially when accompanied by high humidity.
55	Effects of the environmental temperature on *Aedes aegypti*	Insects	2018	B	Level 5: A review on the effect of environmental temperature on *Aedes aegpti* and *Aedes albopictus* mosquitoes.	• Indicates positive associations between increased temperature and adult vector development, disease vector transmission, or

	and *Aedes albopictus* mosquitoes: A review					virus replication and amplification in the vector.<br>• Such associations are particularly identified for VBDs including dengue, chikungunya, West Nile virus, and yellow fever.
56	Medical Entomology: A Textbook on Public Health and Veterinary Problems Caused by Arthropods	Kluwer Academic	2000	C	Others: A textbook with emphasis on arthropod-caused diseases with discussions on epidemiology, vector control, and disease surveillance.	• States that the human biting rate of disease vectors is most frequently estimated by vector landing rates.
57	Mosquito feeding behaviour and how it influences residual malaria transmission across Africa	Proc Natl Acad Sci USA	2019	D	Level 1a: A systematic review to quantify and estimate public health impact of vector biting under different circumstances.	• Indicates that 79% of malaria vector bites occur when people are in bed.
58	Global Change and Human Vulnerability to Vector-Borne Diseases	Clin Microbiol Rev	2004	D	Level 5: A review on the impacts of global changes on VBDs.	• Suggests that the immune status of a population to a particular disease largely influences the sensitivity of a population to a change in exposure.<br>• Indicates that populations which are naïve and nonimmune are of high susceptibility to infectious disease epidemics.
59	The enigmatic reception of DEET - The gold standard of insect repellents	Curr Opin Insect Sci	2014	C	Level 5: A review on the efficacy and usage of insect repellents containing DEET.	• Indicates that repellents significantly reduce mosquito bites and VBD transmission.<br>• Indicates that DEET is capable of reducing mosquito landing and has a distant repelling effect through its potential detection by vector odorant receptors.
60	Fight the Bite for Protection from Malaria Guidelines for DEET Insect Repellent Use	Centres for Disease Control and Prevention	2008	C	Others: A guideline document on the usage of DEET, its properties and precautions.	• Suggests that DEET concentration indicates the length of effectiveness of the product but not necessarily that it is a more efficacious product.<br>• Mentions DEET precautions such as: keeping it out of reach from children, avoiding application to wounds or damaged skin, and avoiding over-application.
61	Effectiveness of a repellent containing DEET and EBAAP for preventing tick bites	Wilderness Environ Med	2002	C	Level 1b: A randomized, double-blind, placebo-controlled study to evaluate the effectiveness of DEET and EBAAP repellents in preventing tick bites.	• Indicates that DEET and EBAAP repellents which are easily applied are moderately effective towards the reduction of tick bite risk.
62	Preventing mosquito and tick bites: A Canadian update	Paediatr Child Heal	2014	C	Level 5: A compilation of available information on tick and insect repellent in	• Suggests the use of synthetic insect repellents containing DEET or Icaridin.

					an attempt to generate recommendations and instructions.	• Suggests that permethrin is another common active ingredient applied to clothing that retains effectiveness for up to six washings.<br>• Indicates that there are alternative 'natural' repellents which are not necessarily safer than DEET or Icaridin and some may not offer adequate protection.<br>• Recommends guidelines on repellent usage such as light application on the basis of it being a chemical which may be harmful if misused. Infants under the age of 6 should be using alternatives to such insect repellents.
63	Tips for using insect repellents	Centre for Health Protection Hong Kong	2018	C	Others: A compilation of available information on the usage of insect repellents and relevant precautions.	• Makes specific remarks on DEET-containing repellent precautions when applied on children, such as the use of roll-on instead of spray repellents.<br>• Remarks that infants under the age of 6 should not use DEET-containing repellents.<br>• Indicates importance of following the product label for guidelines and precautions, regardless of the repellent chosen.
64	The underlying reasons for very high levels of bed net use, and higher malaria infection prevalence among bed net users than non-users in the Tanzanian city of Dar es Salaam: A qualitative study	Malar J	2017	D	Level 4: A cross-sectional, qualitative study on the underlying reasons behind high levels of bed net use as well as higher malaria infection prevalence among bed net users than non-users.	• Provides potential explanations for compromised bet net efficacy and thus higher malaria infection prevalence among bed net users than non-users in other studies, bed net sharing, incomplete bed tucking, the touching of the net while sleeping, and the presence of holes in bed nets.<br>• Indicates collateral benefits of bed net use mentioned by bed net users including the prevention of dust from landing on bed sheets and coverings as well as prevention of household pests.
65	The efficacy of long-lasting nets with declining physical integrity may be compromised in areas with high levels of pyrethroid resistance	Malar J	2013	D	Level 4: A cross-sectional survey on the impact of declining physical integrity in long-lasting insecticide-treated nets on efficacy.	• Indicates that long-lasting insecticide-treated nets with modest hole areas would permit the entrance and feeding of mosquitoes, compromising efficacy.<br>• Suggests that long-lasting insecticide-treated nets are prone to the development of large holes within three years of use, compromising efficacy.

66	The effectiveness of older insecticide-treated bed nets (ITNs) to prevent malaria infection in an area of moderate pyrethroid resistance	Malar J	2020	D	Level 2b: A cohort study evaluating the effectiveness of older insecticide-treated bed nets on preventing malaria infection in moderate pyrethroid resistance areas.	• Indicates no statistically significant findings on the different in infection incidence between users and non-users of the bed nets.<br>• Demonstrates that among insecticide-treated bed net uses, malaria incidence was significantly lower in users of bed nets with no holes compared to those who had bed nets with one hole or more.
67	Insecticide-Treated Bed Nets	Centres for Disease Control and Prevention	2019	D	Others: A guideline document on the usage of insecticide-treated nets and relevant precautions.	• Indicates one type of bed nets which are conventional-treated by dipping them into a mixture of water and insecticide regularly.<br>• Indicates another type of bed nets which are long-lasting insecticide-treated as developed by companies and are associated with sharp decreases in malaria in countries of high bed net coverage.<br>• Concludes that resource mobilisation for procurement of bed nets is a major challenge in the future.
68	A Study on Efficacy of LLINS As Compared To In-Use ITNs Amongst Troops in a Malaria Endemic Area	J Trop Dis	2015	D	Level 2b: An interventional study without randomisation on the comparison of efficacies between long-lasting insecticide-treated nets and insecticide-treated nets in an endemic area.	• Indicates that long-lasting insecticide-treated bed nets are more effective in killing mosquitoes and reducing their Manhour Density compared to insecticide-treated nets.<br>• Indicates that long-lasting insecticide-treated bed nets retained their initial level of effectiveness after washes.<br>• Stresses the importance of effective distribution, usage and retention of long-lasting insecticide-treated bed nets.<br>• Mentions more research needed on insecticide-resistance.
69	Fundamentals of vaccine immunology	Journal of Global Infectious Diseases	2011	E	Level 5: A literature review on vaccine immunology fundamentals.	• Indicates derivation of immunisation from either passive or active means.<br>• Classifies vaccination as a form of active immunisation which exposes unimmunised individuals to a pathogenic agent.<br>• Indicates that vaccines stimulate the immune system to achieve long-term immunity through strong cellular and antibody responses.

						• Concludes that effective immunisation must induce long-term stimulations of both arms of the adaptive immune system.
70	Chemoprophylaxis of tropical infectious diseases	Pharmaceuticals	2010	E	Level 5: A review on the available evidence on chemoprophylaxis of tropical infectious diseases.	• Defines that chemoprophylaxis involves the administration of drug to prevent the development of a disease.<br>• Concludes that chemoprophylaxis should be considered for travellers to high risk areas.<br>• Indicates that in cases where non-drug measures are available to reduce the risk of infection, chemoprophylaxis should play a secondary role.
71	Benefit of Insecticide-Treated Nets, Curtains and Screening on Vector Borne Diseases, Excluding Malaria: A Systematic Review and Meta-analysis	PLoS Negl Trop Dis	2014	D	Level 1a: A systematic review on cluster or individually randomised controlled trials, non-randomised trials with pre- and post-intervention data and rotational design studies to assess the efficacies of insecticide-treated nets, insecticide-treated curtains and insecticide-treated house screening versus no intervention.	• Indicates that insecticide-treated bed nets are highly efficacious in reducing VBD pathogen transmission and morbidity from VBDs with household vector entrance.<br>• Suggests that the prevention of household pests such as rodents and cockroaches is a collateral benefit with the use of insecticide-treated nets.
72	Use of bed nets and factors that influence bed net use among jinuo ethnic minority in southern China	PLoS One	2014	D	Level 4: A cross-sectional study on the extent of and factors that influence bed net use among Jinuo Ethnic Minority.	• Indicates a high bed net availability yet relatively subpar bed net use and coverage.<br>• Suggests a low percentage of correct use of bed nets.<br>• Indicates that higher household income was associated with higher bed net use, and unaffordability was a main issue hindering bed net purchase.<br>• Indicates that house types with traditional wood walls and terracotta roofs, and head of households who knew more about bed net efficacy were more likely to use bed nets.<br>• Suggests that the hanging of nets was considered troublesome by many surveyed.
73	Population preference of net texture prior to bed net trial in Kala-Azar-endemic areas	PLoS Negl Trop Dis	2007	D	Level 4: A cross-sectional study on the population preference of long-lasting insecticidal net texture before starting a community-based efficacy trial of long-lasting insecticidal nets.	• Indicates that users had a slight preference for bed nets made of polyester over that made of polyethylene due to relatively greater softness.<br>• Indicates that majority of long-lasting insecticidal net users in Nepal and India hung their nets with ropes and placed their nets on sticks respectively.

						• Concludes that the best tool of intervention would be light-blue, rectangular polyester long-lasting insecticidal nets of different sizes.
74	Plant-based insect repellents: A review of their efficacy, development and testing	Malar J	2011	C	Level 5: A review on the testing, efficacy, and safety of plant-based insect repellents.	• Indicates that Eucalyptus has proven clinical efficacy against malaria and is the only plant-based chemical advocated for use in disease endemic areas by CDC.<br>• Indicates that Citronella is considered effective to protect from host-seeking mosquitoes, but only for a short duration of around two hours.<br>• Suggests that despite a potential of mosquito bite protection, conflicting findings are available on the efficacy of neem oil, and is not recommended for travellers in disease endemic areas.<br>• Mentions that unlike DEET which has undergone stringent testing and generally has a good safety profile, plant-based repellents are not as rigorously tested. Neem, for instance, may cause dermatitis when used undiluted.
75	Eucalyptus essential oil as a natural pesticide	For Ecol Manage	2008	C	Level 5: A review on the use of eucalyptus oil as a natural pesticide.	• Indicates that Quwenling, a Eucalyptus-based product, provides protection against *Anopheles* mosquitoes with efficacy parallel to DEET.<br>• Indicates that lemon eucalyptus oil and PMD are two plant-based insect-repellents approved by the USEPA for protection against mosquitoes.<br>• Suggests that eucalyptus oil has potential for environmentally benign pest control unlike synthetic pesticides which have environmental and toxicological implications.
76	Severe allergic reaction to diethyltoluamide (DEET) containing insect repellent	Allergy, Asthma Clin Immunol	2014	C	Level 5: A case study on the potential severe allergic reaction to DEET-containing insect repellent.	• Suggests that DEET-containing insect repellents may induce severe cutaneous allergic reactions, and may further put such patients at risk of anaphylaxis with future exposure.
77	Infectious Disease Epidemiology: Theory and Practice	Am J Epidemiol	2007	D	Level 5: A book reviewing infectious disease epidemiology.	• States that untreated bed nets are estimated to provide half the protective effect of insecticide-treated bed nets, and serves as merely a physical barrier.

						• Indicates that insecticide-treated bed nets provide an additional chemical barrier to repel disease vectors.
78	Airflow attenuation and bed net utilization: Observations from Africa and Asia	Malar J	2012	D	Level 5: An observational study to assess the effect of bed nets on temperature, humidity and airflow in rural homes in Asia and Africa, thus further evaluate the associated thermal discomfort of users.	• Suggests that bed nets reduce airflow compared to unattenuated airflow, and may limit bed net use.<br>• Suggests that thermal discomfort associated with bed nets is most problematic during hot and humid climates, which is coincidentally the peak of malaria vector densities and pathogen transmission force.<br>• Indicates no statistically significant different in temperature or humidity between the inside and outside of the bed net.<br>• Suggests that higher bed net mesh size is correlated with decreased air flow.<br>• Encourages better designs to overcome such limitations.
79	Reported reasons for not using a mosquito net when one is available: A review of the published literature	Malar J	2011	D	Level 5: A literature review on the barriers to mosquito net use in malaria-pandemic countries through looking into published research on self-reported reasons.	• Indicates that thermal discomfort due to heat and perceived low mosquito density (complacency) are the two major identified reasons for non-use.<br>• Mentions technical factors related to mosquito net use (unable or inconvenient to wash or hang mosquito net) as a reason for non-use.
80	Assessing the effective use of mosquito nets in the prevention of malaria in some parts of Mezam division, Northwest Region Cameroon	Malar J	2016	D	Level 4: A laboratory study and cross-sectional questionnaire to assess the acceptability and efficacy of long-lasting insecticide-treated bed nets on malaria prevalence.	• Indicates statistically significant malaria prevalence reduction among users of long-lasting insecticide-treated bed nets compared to non-users.<br>• Shows that half of the respondents considered heat and the feeling of suffocation as the major problem with long-lasting insecticide-treated bed net use.<br>• Reinforces the importance of encouraging utilisation of bed nets to match ownership, as well as the continuous and consistent free distribution of bed nets to vulnerable groups.
81	Factors influencing vaccine acceptance and hesitancy in three informal settlements in Lusaka, Zambia	Vaccine	2018	E	Level 5: A qualitative study (focus group) on factors influencing vaccine acceptance and hesitancy in three informal settlements in Lusaka, Zambia.	• Indicates alcohol, religious prayers, and traditional remedies as community-perceived alternatives to vaccine.

						• Demonstrates that fear of injections (needle phobia), perceived risk of infection under vaccination, traditional religious beliefs, low perceived need for immunisation (complacency), lack of knowledge and confidence in vaccine mechanisms and efficacies are common perceptions that limit immunisation coverage in communities.<br>• Encourages education and better research on community vaccination preferences to improve immunisation coverage.<br>• Suggests mobile immunisation campaigns to reach areas with reduced access.
82	Risk factors of underutilization of childhood immunizations in ultraorthodox Jewish communities in Israel despite high access to health care services	Vaccine	2012	E	Level 4: A cross-sectional study to assess immunization status of children from ultraorthodox Jewish communities and associating such with socio-demographic factors and parents' attitudes regarding vaccines.	• Suggests that vaccine underutilisation in the studied community was significantly associated with factors such as parental religious beliefs against vaccination, perceived risk of vaccine preventable diseases as low, and having more than 6 siblings.<br>• Encourages health education on the risks of vaccine preventable diseases and confidence enhancement in the Ministry of Health to modify the under-immunisation risk factors.
83	Malaria vaccination: a major milestone	Lancet Infect Dis	2019	E	Level 5: An editorial on the overall developmental progress of malaria vaccination and milestones in malaria control.	• Mentions the launch of the pilot RTS,S vaccination program as a potential prophylactic strategy against malaria.<br>• Indicates that the phase 3 clinical trial of RTS,S was large-scale and demonstrated that four in ten cases of malaria can be prevented.<br>• Mentions the integration of the RTS,S vaccine into the national immunisation programs of Malawi, Ghana and Kenya over the next 5 years, in hope of decreasing malaria mortalities.<br>• Mentions that accessibility to all four doses of the vaccine for maximum protection is a challenge to the feasibility of the intervention.<br>• Suggests importance of top-down coordination from government bodies down to community level.

						• Emphasises that RTS,S only offers partial protection and is thus only a supplementary primary prevention strategy.
84	Barriers to effective uptake and provision of immunization in a rural district in Uganda	PLoS One	2019	E	Level 4: A cross-sectional mixed methods study to evaluate immunisation services and identify the gaps in health systems that contribute as barriers to effective uptake and provision of immunisation in Uganda.	• Indicates limited access to immunisation centres due to poor road terrain, in turn affecting program effectiveness and the timely delivery of supplies such as refrigerator gas and vaccines.<br>• Suggests need for improvement in immunisation services in areas including vaccine supply, service delivery point expansion, more health workers, and better transport and tailored mechanisms.
85	The efficacy of some commercially available insect repellents for *Aedes aegypti* (*Diptera: Culicidae*) and *Aedes albopictus (Diptera: Culicidae)*	J Insect Sci	2015	C	Level 4: An interventional study to evaluate and compare the efficacies of some commercially-available insect repellents.	• Indicates that regarding the two mosquito species tested, *Ae. Albopictus* were generally less attracted to the subject's hand compared to *Ae. Aegypti.*<br>• Suggests that different active ingredients in commercially available mosquito repellent products vary in terms of their duration and strength of repellence.<br>• Indicates that citronella, a plant-based repellent, did not have any significant repellence effect.<br>• Concludes that DEET-containing products have more long-lasting repellent effects.
86	The insect repellent N,N-diethyl-m-Toluamide (DEET) induces angiogenesis via allosteric modulation of the M3 muscarinic receptor in endothelial cells	Sci Rep	2016	C	Level 5: A laboratory study to investigate the potential of angiogenesis induction of DEET and associated health risks.	• Demonstrates that DEET stimulates endothelial cells and promotes angiogenesis, in turn increasing tumour growth.<br>• Encourages risk of assessment of DEET in humans to provide safe conditions of use.
87	Is DEET a dangerous neurotoxicant?	Pest Manag Sci	2019	C	Level 5: A review on the safety of DEET when used as an insect repellent.	• Indicates that while some studies reflect that DEET poses minor health risks from human use, a large analyses from the US Poison Centre has concluded that DEET presents little to no risk when applied according to product labels.<br>• Suggests that the number of reports that document serious health effects after proper use of DEET is minimal.

						• Expresses that continued and proper use of DEET is essential for human health.
88	DEET-based insect repellents: Safety implications for children and pregnant and lactating women	Can Med Assoc J	2003	C	Level 5: A review on the available evidence on the effectiveness and safety of DEET-based products, especially in young children and pregnant or lactating women.	• Indicates a lack of existing evidence that demonstrates that young children are more prone than adults to the neurotoxic effects of DEET.<br>• Indicates there is no evidence on the health hazards of DEET use by pregnant or lactating women to unborn babies or breast-fed children.<br>• Mentions that limited studies demonstrate how the DEET concentration positively relates to the protection times of insect repellents.
89	Insecticide-treated Bednets to Control Dengue Vectors: Preliminary Evidence From a Controlled Trial in Haiti	Trop Med Int Heal	2008	D	Level 1b: A cluster-randomized trial in Haiti to test the efficacy of insecticide treated bed nets.	• Indicates that the usage of insecticide treated bed nets was associated with reduction of dengue vector populations.<br>• Mentions that such effect was seen in the long-term as well.
90	The effect of malaria control on Plasmodium falciparum in Africa between 2000 and 2015	Nature	2015	D	Level 3a: A review and meta-analysis on the trends from 2000 to 2015 and the quantification of attributable effects of malaria disease control efforts.	• Concludes insecticide-treated nets as the most widespread intervention that adverted 68% of clinical malaria cases.<br>• Encourages increased access to relevant control interventions as well as effectiveness maintenance under emergence of insecticide and drug resistance.<br>• Suggests that indoor residual spraying and prompt treatment of malaria cases with artemisinin-based combination therapy also contribute to reduced malaria prevalence when implemented at scale.
91	Community-wide effects of permethrin-treated bed nets on child mortality and malaria morbidity in western Kenya	Am J Trop Med Hyg	2003	D	Level 1b: A randomised controlled trial to evaluate the effect of permethrin-treated bed nets on child mortality and malaria morbidity in West Kenya.	• Demonstrates that insecticide-treated net use in nearby compounds had a protective effect for child mortality, moderate anaemia, high-density parasitaemia, and haemoglobin levels in compounds lacking the bed nets.<br>• Suggests that the community effect of widespread bed net coverage on nearby compounds without nets is potentially equivalent to that observed in villages with insecticide-treated bed net use.

						• Reinforces importance of high coverage with insecticide-treated bed nets to maximise public health impact.
92	Preventing childhood malaria in Africa by protecting adults from mosquitoes with insecticide-treated nets	PLoS Med	2007	D	Level 5: A model-based study to assess and estimate insecticide-treated net coverage thresholds for entire populations to provide equivalent individual- and community-level protection from disease vectors.	• Indicates that high (80% use) of insecticide-treated nets to exclusive target young children and pregnant women delivers limited protection for these vulnerable groups.<br>• Indicates that relatively modest coverage of all adults and children can achieve more equitable community-wide benefits equivalent to or greater than personal protection.
93	The ability of *Anopheles gambiae* mosquitoes to bite through a permethrin-treated net and the consequences for their fitness	Sci Rep	2019	D	Level 5: An entomological research to assess the ability of an insecticide-sensitive strain of *Anopheles gambias* to bite through a permethrin-treated or untreated net and their subsequent survival and fertility.	• Indicates that a majority of mosquitoes took blood through the insecticide-treated net despite permethrin irritancy.<br>• Suggests that insecticide treatment reduced the biting time, blood-meal size and fertility of the mosquitoes, killing 15% of the mosquitoes within 24 hours of feeding, compared to no treatment of the bed nets.<br>• Mentions that irritancy and toxicity of insecticide-treated nets to mosquitoes may potentially be reduced when mosquitoes contact and feed on bed net users.
94	Vaccines and vaccination against yellow fever - WHO Position Paper	WHO	2013	E	Others: A WHO position paper on vaccination against yellow fever.	• Mentions that while no human efficacy studies have been performed with the yellow fever vaccine, observations such as the absence of laboratory-associated infections in vaccinated workers and the occurrence of yellow fever only in unvaccinated people in Brazil and other South American countries following initial use support the protective effect of the vaccine.<br>• Indicates that a single dose of yellow fever vaccine would suffice for a sustained life-long protective immunity against yellow fever disease.
95	Review article: Efficacy and duration of immunity after yellow fever vaccination: Systematic review on the need for a booster every 10 years	Am J Trop Med Hyg	2013	E	Level 2a: A systematic review on the protective efficacy and duration of immunity of yellow fever vaccine in residents living in and travelers to disease-endemic areas, in turn assessment of the	• Concludes that a single dose of yellow fever vaccine is highly efficacious and immunogenic, providing sustained life-long protective immunity against yellow fever.

					need for a booster for the yellow fever vaccine.	• Suggests that a booster dose of yellow fever vaccine is not necessary.
96	Vaccines against tick-borne encephalitis: WHO position paper	WHO	2011	E	Others: A WHO position paper on vaccination against tick-borne encephalitis.	• Mentions that immunisation provides the most effective protection against tick-borne encephalitis.<br>• Mentions that all 4 vaccines manufactured by Austria (1), Germany (1) and the Russian Federation (2) are safe and efficacious for adults.<br>• Mentions that the available vaccines efficaciously protect against all tick-borne encephalitis virus subtypes in Asian and European endemic areas.
97	Tick-borne encephalitis: A review of epidemiology, clinical characteristics, and management	World J Clin Cases	2015	E	Level 5: A review on the epidemiology, clinical characteristics and management of tick-borne encephalitis.	• Mentions a growing threat and increasing incidence of the disease, especially in all endemic European and Asian countries.<br>• Mentions clinical manifestations of the disease including mild meningitis to severe meningoencephalitis with or without paralysis.<br>• Indicates that while there is no specific antiviral treatment for the disease, vaccination is efficacious to prevent the disease for at-risk populations and persons visiting endemic areas.
98	Japanese Encephalitis Vaccines: WHO position paper	WHO	2015	E	Others: A WHO position paper on vaccination against Japanese encephalitis.	• Mentions that vaccinated populations demonstrate a significant reduction in Japanese encephalitis cases.<br>• Recommends the integration of Japanese encephalitis vaccination into national immunisation schedules in all areas affected by the disease.<br>• Indicates that high Japanese encephalitis vaccination coverage should be achieved and sustained in at-risk populations.
99	Japanese encephalitis vaccines: Immunogenicity, protective efficacy, effectiveness, and impact on the burden of disease	Hum Vaccines Immunother	2017	E	Level 5: A review on the immunogenicity and protective ability of different Japanese encephalitis vaccines available as well as their impact in reducing disease burden in endemic countries.	• Indicates that all marketed vaccines which belong to the Japanese encephalitis virus genotype III are efficacious against other genotypes and strains.<br>• Suggests that the general protective response for the Japanese encephalitis vaccines last for

						three or more years, depending on the type and number of doses of the vaccine.
100	Malaria vaccine pilot launched in Malawi	WHO	2019	E	Others: A news release on the launch of a malaria vaccine pilot in Malawi.	• Mentions the launch of the pilot RTS,S vaccination program that requires four doses as a potential prophylactic strategy against malaria.<br>• Indicates that the phase 3 clinical trial of RTS,S was large-scale and demonstrated that four in ten cases of malaria can be prevented.<br>• Mentions the launch of the malaria vaccine pilot in Malawi, followed by Ghana and Kenya over the next 5 years, in hope of decreasing malaria mortalities.
101	Long-term use of antimalarial drugs in rheumatic diseases	Clin Exp Rheumatol	2012	E	Level 4: A case series to assess the long-term use of antimalarial drugs and potential causes of discontinuation.	• Suggests potential health risks and adverse events of antimalarials that prompt discontinuation, such as gastrointestinal, neuro-psychiatric and skin problems, as well as ophthalmologic adverse events like antimalarial retinopathy and rare irreversible loss of vision.<br>• Suggests that antimalarials have a good balance between benefit and risk.<br>• Emphasises on importance of ophthalmologic monitoring to look into the potential for rare but severe ophthalmologic toxicities when taking antimalarials.
102	Prophylaxis of Malaria	Mediterr J Hematol Infect Dis	2012	E	Level 5: A review on the nature, types, and potential barriers or problems that may arise from malaria chemoprophylaxis.	• Indicates that potential challenges to malaria chemoprophylaxis include barriers such as high costs, adverse reactions to medications and fatalities.<br>• Recommends a revised guideline for chemoprophylaxis use by travellers in low risk areas.
103	Prevention of malaria in long-term travellers	J Am Med Assoc	2006	E	Level 3a: A systematic review on the risk of malaria in long-term travellers, recent developments in personal protective measures, safety and tolerability of malaria chemoprophylaxis during long-term use, and evaluation of relevant prevention strategies.	• Suggests that long-term travellers are at a higher risk of malaria infection than short-term travellers.<br>• Indicates that personal protective measures and chemoprophylaxis are not adequately used in long-term travellers.<br>• Proposes strategies such as the discontinuation of chemoprophylaxis after the initial period

						and sequential regimens with different medications for chemoprophylaxis.<br>• Points out that vivax malaria relapses cannot be prevented with current first-line chemoprophylaxis regimens.<br>• Recommends individualised guidelines for malaria prevention in long-term travellers.
104	First FDA-approved vaccine for the prevention of dengue disease in endemic regions	The U.S. Food and Drug Administration	2019	E	Others: A news release on the first FDA-approved vaccine for dengue prevention in endemic regions.	• Mentions that Dengvaxia, the dengue vaccine, is believed to be 76% effective in targeting all dengue virus serotypes in people ages 9-16 who have laboratory-confirmed previous dengue infection and live in endemic areas.<br>• Mentions that the second infection with dengue is often much more severe than the first, with the potential of developing dengue haemorrhagic fever (DHF).<br>• Mentions that Dengvaxia is not approved for use in individuals not previously infected by any dengue virus serotype since it would be similar to a first dengue infection causing subsequent dengue infections to result in severe dengue disease.
105	Systematic review of dengue vaccine efficacy	BMC Infect Dis	2019	E	Level 1a: A systematic review on the efficacy of dengue vaccine.	• Concludes that compared with other commercially available vaccines, Dengvaxia, the only U.S. Food and Drug Administration (FDA)-approved vaccine against dengue fever, demonstrated poor efficacy.<br>• Mentions that dengue vaccine may increase the risk of severe dengue symptoms in seronegative patients infected for the first time after vaccination.
106	Aedes mosquitoes acquire and transmit Zika virus by breeding in contaminated aquatic environments	Nat Commun	2019	G	Level 5: An animal (mouse) experimental investigation into the acquirement and transmission of Zika virus due to breeding in aquatic environments, such as urine and sewages.	• Indicates that urine could be a source of Zika virus and has the potential for transmission to the vector during breeding.
107	Aedes–Chikungunya Virus Interaction: Key Role of Vector Midguts Microbiota and Its Saliva in the Host Infection	Front Microbiol	2019	G	Level 5: A literature review on Aedes-Chikungunya virus interaction.	• Suggests that *Aedes* females are capable of transmitting the virus.<br>• Indicates that mosquito microbiota is important to virus infection in the mosquito vector.

						• Suggests that *Aedes* saliva contains substances that can affect the host's viral responses.
108	Characterization of larval habitats for *Anopheline* mosquitoes in a malarious area under elimination program in the southeast of Iran	Asian Pac J Trop Biomed	2014	G	Level 5: An entomological study to evaluate the influence of environmental characteristics of various larval habitats on the subsequent prevalence of *Anopheline* mosquitoes.	• Suggests that most prominent larval habitats were still water and water bodies such as riverbeds.
109	A review and framework for understanding the potential impact of poor solid waste management on health in developing countries	Arch Public Heal	2016	G, H	Level 5: A review to present a framework to understand the correlation between insufficient solid waste management and health outcomes.	• Illustrates the burden imposed on health by poor waste management.<br>• Demonstrates clear linkages between poor solid waste management and health consequences.<br>• Suggests that the determined linkages can guide future research and policy.
110	Lethal effects of short-wavelength visible light on insects	Sci Rep	2014	F	Level 5: An entomological study to assess how the toxicity imposed by blue light varies with the development of insects.	• Indicates that blue light is lethal towards insects such as mosquitoes.<br>• Suggests that certain insects such as *D. Melanogaster* have variable susceptibility to different wavelengths across different developmental stages.<br>• Mentions that blue light irradiation can effectively be used in pest control via wavelength adjustments to match susceptibility with the developmental stage of the insect.
111	Solid-waste management in Jalandhar city and its impact on community health	Indian J Occup Environ Med	2008	H, J	Level 4: A cross-sectional study on solid waste management measures to find out their linkage with the prevalence of vector-borne diseases in order to generate relevant recommendations.	• Demonstrates linkages between solid waste accumulation and a higher degree of vector-borne infections.<br>• Concludes with recommendations to modernize management practices such as through waste separation.<br>• Encourages health education to increase public awareness and improve vector control programmes.
112	Microbial pathogens of public health significance in waste dumps and common sites	J Environ Biol	2007	H	Level 5: A study collecting soil samples from dumping sites to determine the presence of microbial pathogens.	• Indicates that several bacterial species were detected which have the potential to cause infections and eventual mortality.<br>• Mentions that waste management issues should be addressed imminently.

113	Evaluation of a comprehensive slip, trip and fall prevention programme for hospital employees	Ergonomics	2008	G	Level 2b: A 10-year longitudinal cohort study to determine the various slip, trip and fall (STP) incidents amongst hospital workers and to evaluate the efficacy of a prevention program.	• Indicates that the major cause of STFs was due to liquid means e.g. water, fluid, slippery surfaces.<br>• Mentions that STF incidents were deemed as preventable through prevention and education programmes.
114	A spreading concern: Inhalational health effects of mould	Environ Health Perspect	2007	G	Level 5: A review on the issues surrounding mould contamination and the risks arising from exposure.	• Mentions that many sources indicate health hazards and illnesses being linked with mould exposure.<br>• Indicates that further information and details regarding the health risks is very limited.
115	Health effects associated with the disposal of solid waste in landfills and incinerators in populations living in surrounding areas: A systematic review	Int J Public Health	2013	H	Level 2a: A systematic review on the health hazards and impacts for those living near landfills.	• Suggests risks of congenital anomalies and respiratory conditions when residing near special waste landfills.
116	Waste mismanagement in developing countries: A review of global issues	Int J Environ Res Public Health	2019	G, H	Level 5: A review on the mismanagement of waste in developing countries.	• Identifies key issues associated with waste mismanagement.<br>• Identifies three levels of impacts with regard to waste mismanagement: local impacts e.g. spread of diseases due to vectors; regional impacts e.g. due to water body pollution; global impacts: e.g. global warming.
117	Community mobilization and household level waste management for dengue vector control in Gampaha district of Sri Lanka; an intervention study	Pathog Glob Health	2012	H	Level 2b: An interventional study involving programmes in areas such as household waste management and raising awareness on the importance of SWM for dengue control.	• Indicates that to achieve high efficacy and sustainable dengue control, coordination between local institutions and heightened household responsibility are crucial.
118	SDG7: Data and Projections	International Energy Agency	2019	F	Others: A report on the access to electricity and solutions.	• Concludes on figures such as the number of people with access to clean cooking and electricity.
119	A Simple Non-Powered Passive Trap for the Collection of Mosquitoes for Arbovirus Surveillance	J Med Entomol	2013	F	Level 5: An entomological study to evaluate the effectiveness of various non-powered passive traps as alternatives to power-requiring light traps.	• Indicates that several passive traps were inexpensive and showed efficacy.<br>• Suggests that non-powered passive traps may be suitable for mosquito collection and pathogen surveillance.
120	Are Yellow Sticky Traps an Effective Method for Control of Sweetpotato Whitefly,	J Insect Sci	2012	F	Level 1b: A randomised controlled trial to assess the effect of sticky traps on the	• Indicates that the density of whiteflies was reduced in greenhouses, as compared to greenhouses without traps.

	Bemisia tabaci , in the Greenhouse or Field?				population of *Bemisia tabaci* in the greenhouse and field.	• Indicates that the density of whiteflies was not reduced when sticky traps were used in the field.<br>• Suggests that sticky traps may be an effective method to reduce whitefly population in greenhouses, but not in fields.
121	Understanding Water Storage Practices of Urban Residents of an Endemic Dengue Area in Colombia: Perceptions, Rationale and Socio-Demographic Characteristics	Plos One	2015	G	Level 2b: An interventional study (cluster randomized trial) to understand water storage practices in a selected population and the linkage to socio-demographic characteristics.	• Indicates that water storage was common due to two core reasons: concerns of scarcity and perceived high prices in water rates.<br>• Mentions that understanding such social practices, for example what type of social dynamic result in water storage practice, is critical to mitigate the risk of *Ae. Aegypti* breeding.
122	A qualitative study of community perception and acceptance of biological larviciding for malaria mosquito control in rural Burkina Faso	BMC Public Health	2018	G	Level 5: A qualitative study (focus group) to explore community perceptions towards the usage of larvicides for mosquito control.	• Suggests that modern day larvicides are effective and have low health risks.<br>• Mentions that the biological larviciding programme produced large amounts of positive reviews.<br>• Indicates that the high acceptance of larviciding programs shows potential for long-term routine implementation.
123	Barriers to effective municipal solid waste management in a rapidly urbanizing area in Thailand	Int J Environ Res Public Health	2017	H	Level 5: A qualitative study (in-depth interview) with interviews conducted to determine the major factors influencing waste management in a selected municipality.	• Mentions that influencing factors or barriers to waste management included socio-cultural, technical, financial, organizational, and legal-political barriers as well as population growth.<br>• Suggests that external support from institutions can enhance the opportunities to improve the situation surround waste programmes and management.
124	Insect Vision: Ultraviolet, Colour, and LED Light	University of Georgia Department of Entomology	2011	F	Level 5: A review on insect vision with focus on the types of light radiation that attract insect and the reason behind the attraction.	• Indicates that insects can detect UV and colours via their photoreceptors.<br>• Mentions that for many insects, their attraction to UV is well-evidenced.<br>• Suggests that different colours of light have varying efficacies in attracting different insects.
125	Mosquito Attraction: Crucial Role of Carbon Dioxide in Formulation of a Five-	J Chem Ecol	2015	F	Level 5: An entomological study to determine behavioural responses of the malaria mosquito (Anopheles coluzzii) to	• Indicates that in the ultimate five-component blend, carbon dioxide was involved and was determined to have a crucial role in increasing the attractiveness of the blend.

	Component Blend of Human-Derived Volatiles				various carbon dioxide containing formulations of volatiles.	• Suggests that the five component blend, involving carbon dioxide, can potentially be used for mass trapping of malaria vectors and subsequent reduction of mosquito biting.
126	Traps for Capturing Insects	Encyclopaedia of Entomology	2008	F	Others: A review article on insect capturing traps exploring the different types and the mechanisms behind attraction.	• Mentions that trapping systems may be used for the purpose of biological control.<br>• Mentions that octenol is an example of a naturally-emitted chemical (from oxen and cows), which when combined with carbon dioxide has efficacy in attracting mosquitoes.
127	Carbon dioxide, odorants, heat and visible cues affect wild mosquito landing in open spaces	Front Behav Neurosci	2018	F	Level 5: An entomological experiment on wild mosquitoes in an open space to determine how carbon dioxide, odorants, heat and visible cues may affect mosquito attraction and landing.	• Indicates that mosquitoes were not caught in the absence of heat.<br>• Suggests that humidification may affect the distance upon which mosquito attraction occurs but did not influence landings.<br>• Concludes that compared to odorants, humidity and black colour, the presence of carbon dioxide and heat showed greater effects on mosquito landings.
128	Reduced Incidence of Chikungunya Virus Infection in Communities with Ongoing Aedes Aegypti Mosquito Trap Intervention Studies — Salinas and Guayama, Puerto Rico, November 2015–February 2016	Morb Mortal Wkly Rep	2016	F	Others: A USCDC weekly report on a ongoing study to investigate the efficacy of Aedes Aegypti Mosquito Trap traps on the incidence of Chikungunya virus infection	• Indicates a lower incidence of virus infection in the intervention community when compared to the non-intervention community.<br>• Suggests that the AGO traps can reduce transmission of VBDs by reducing the vector density.
129	Comparative efficacy of small commercial traps for the capture of adult *Phlebotomus papatasi*	J Vector Ecol	2011	F	Level 5: An entomological study on ten commercial mosquito traps to assess their efficacy towards the capturing of Adult *Phlebotomus Papatasi*.	• Indicates that some mosquito traps performed extremely poorly.<br>• Concludes that some traps that were effective towards the capture of mosquitoes had similar effects towards sand flies.
130	Ultraviolet safety assessments of insect light traps	J Occup Environ Hyg	2016	F	Level 5: A laboratory research to determine the risk of UV radiation exposure from various insect light traps.	• Suggests that human exposure to UV light under insect light trap usage was significantly lower than the guidelines for maximal exposure.<br>• Mentions that there is a need to revisit 'hazard' distances on insect light trap labels as these may be raising unnecessary concerns and may be counter-intuitive towards good

						public health practice as a result of an overestimation of usage risks.
131	Killing of flies in electrocuting insect traps releases bacteria and viruses	Curr Microbiol	2000	F	Level 5: An experimental study where the release of particles and bacteria was assayed upon the electrocution of flies using electrocuting insect traps.	• Indicates that electrocuting insect traps could potentially lead to the spread of infectious diseases agents upon electrocution.<br>• Mentions that the above conclusion, however, depends on the route of contamination for the particular insect.
132	Breeding sites of *Aedes aegypti*: Potential dengue vectors in dire Dawa, east Ethiopia	Interdiscip Perspect Infect Dis	2015	G	Level 5: An entomological study with an entomological survey to assess the presence of dengue vectors in tires which have been discarded as well as artificial water containers in selected houses and peridomestic locations.	• Indicates that *Aedes aegypti* are found to breed in a large range of artificial containers.<br>• Suggests that the presence of water may be the most important factor to determine mosquito breeding.<br>• Indicates the need for further investigation to evaluate whether the breeding mosquitoes are capable of transmitting disease.<br>• Suggests that water chemistry may influence the risk of breeding.
133	UNICEF Djibouti	The United Nations Children's Fund	2019	H	Others: A Djibouti situation report on the heavy rains and floods in 2019.	• Highlights that families affected by the floods are exposed to greater risks of morbidity and mortality as a result of water-borne disease and VBD risks.
134	Solid Wastes Provide Breeding Sites, Burrows, and Food for Biological Disease Vectors, and Urban Zoonotic Reservoirs: A Call to Action for Solutions-Based Research	Front Public Heal	2020	G	Level 5: A literature review to identify gaps in research and pollution to the vicious cycle associated with solid wastes which involves disease vectors, infection and disease.	• Mentions that VBDs are associated with solid waste accumulation.<br>• Suggests that vectors such as the *Aedes* species frequently breed in areas such as garbage dumps.<br>• Indicates that the management of solid waste is crucial in order to prevent development of environments which are favourable to vectors such as the *Aedes* species.
135	Household wastes as larval habitats of dengue vectors: Comparison between urban and rural areas of Kolkata, India	PLoS One	2015	G	Level 5: An entomological study to investigate the relationship between household wastes such as porcelain and plastic wastes and larval habitats of dengue vectors.	• Indicates that porcelain and plastic wastes are correlated with a higher larval productivity.<br>• Suggests that household waste linkage to the productivity of *Aedes* is a risk factor for dengue epidemics.
136	Chemical signalling and insect attraction is a conserved trait in yeasts	Ecol Evol	2018	J	Level 2b: A cohort study on the attraction of insects towards different yeasts.	• Concludes that the flies studied have attraction towards all nine yeasts, which corroborates past research on the mutual interactions occurring between insects and yeasts.

137	Chemical Cues that Guide Female Reproduction in *Drosophila melanogaster*	J Chem Ecol	2018	J	Level 5: A review on vector attraction to chemicals.	• Mentions that flies such as *Drosophila* are attracted to and feed on both microbial communities infesting fruits as well as decomposed fruits.<br>• Mentions that other flies such as *D. Melanogaster* similarly are attracted to decomposed fruits and the yeast that grows on the fruits.<br>• Suggests that such 'chemicals' are important as sources of communication amongst vectors and influence their egg-laying and reproductive behaviours.
138	Environmental and health impacts of household solid waste handling and disposal practices in Third World cities: The case of the Accra Metropolitan Area, Ghana	J Environ Health	2005	J	Level 4: A cross-sectional study relating the incidence of diseases such as diarrhoea and respiratory infections to the household solid waste management practices.	• Mentions that associations have been found between water storage practice and the presence of kitchen houseflies, as well as between houseflies in the kitchen and diarrhoea incidence in children.<br>• Concludes that improper waste management can be a breeding ground for pathogenic organisms.
139	Role of Flies as Vectors of Foodborne Pathogens in Rural Areas	ISRN Microbiol	2013	J	Level 5: An entomological research to explore the presence of various foodborne pathogens in flies collected from different sites.	• Demonstrates that vectors are capable of transmitting foodborne pathogens.<br>• Mentions that the transmission of foodborne pathogens and potential disease by flies is higher in those that were collected in habitats closer to areas of animal production.
140	Information to act: Household characteristics are predictors of domestic infestation with the Chagas vector *Triatoma dimidiata* in central America	Am J Trop Med Hyg	2015	I	Level 4: A cross-sectional study to evaluate the risk factors for household infestation with *Triatoma dimidata*.	• Mentions that amongst other risk factors, insects were commonly found where there were cracks and crevices in the walls.<br>• Concludes that rustic household conditions are the most significant factor in predicting infestation.<br>• Suggests household improvement as a key strategy to counteract *Triatoma dimidiata* infestation.
141	New prototype screened doors and windows for excluding mosquitoes from houses: A pilot study in rural Gambia	Am J Trop Med Hyg	2018	I	Level 1b: A randomized controlled trial (household) on the efficacy of four different prototype screens doors and windows in preventing mosquito entry, as well as evaluating characteristics such as durability of the prototypes.	• Indicates that all of the doors were effective in reducing mosquito entry as compared to the control houses.<br>• Indicates that ten weeks after installation most of the doors and windows remained in good condition.

						• Suggests that screens and windows may be effective measures to reduce mosquito entry and vector exposure without the need for ongoing maintenance.
142	House Screening With Insecticide-Treated Netting Provides Sustained Reductions in Domestic Populations of Aedes Aegypti in Merida, Mexico	PLoS Negl Trop Dis	2018	I	Level 1b: A cluster randomised controlled trial to test the efficacy of insecticide-treated nettings in reducing *Aedes Aegypti* populations.	• Indicates that the usage of house screening was associated with reduced presence and abundance of the vector.<br>• Suggests house screening as a feasible approach to reduce vector presence.<br>• Recommends larger trials.
143	The evidence for improving housing to reduce malaria: A systematic review and meta-analysis	Malar J	2015	I	Level 1a: A systematic review using various studies to evaluate the hypothesis that improvement in housing can effectively reduce malaria incidence by decreasing vector household entry.	• Mentions that while evidence is arguably inadequate, consistency still indicates that house conditions are a significant risk factor for malaria.<br>• Mentions that there are studies on the benefits of house screenings, closing eaves and other household modifications. However, more research needed to assess protective effect of these specific household features individually.
144	The effect of screening doors and windows on indoor density of *Anopheles arabiensis* in south-west Ethiopia: A randomized trial	Malar J	2013	I	Level 1b: A randomised controlled trial assessing the effects of the introduction of metal mesh screening towards reduction of *Anopheles arabienis* indoor density.	• Indicates that the screening of both doors and windows achieved reduced indoor density of *Anopheles arabienis*.<br>• Considers door and window screen installation as a cheap and easy vector control strategy.
145	Integrated approach to malaria prevention at household level in rural communities in Uganda: Experiences from a pilot project	Malar J	2013	I	Level 4: A cross-sectional study to examine the effectiveness of a pilot project promoted prevention of malaria at household level.	• Reports that demonstration households, where measures such as the closing and screening of windows were strictly adopted, had fewer mosquitoes in the house.
146	Management of rodent exposure and allergy in the paediatric population	Curr Allergy Asthma Rep	2013	I	Level 5: A review of literature on rodents and their associated allergens, as well as the effects of exposure.	• Mentions that allergens arising from rodents are a key cause of asthma morbidity in children.<br>• Suggests that the above and other risks can be significantly reduced through pest management measures.<br>• Indicates that amongst these measures, dealing with entry points or cracks is considered an important housekeeping practice to minimise rodent entrance.

147	Experimental evaluation of rodent exclusion methods to reduce hantavirus transmission to residents in a Native American community in New Mexico	Vector Borne Zoonotic Dis	2002	I	Level 4: An interventional study to evaluate the effectiveness of rodent proofing to reduce hantavirus transmission in a selected community.	• Mentions that rodent proofing of homes (such as sealing openings and repairing screens and windows) collectively contributed to a reduction in rodent abundance.<br>• Infers that effectiveness towards rodent proofing methods thereby can reduce the risk of hantavirus transmission.
148	Use and acceptance of long lasting insecticidal net screens for dengue prevention in Acapulco, Guerrero, Mexico	BMC Public Health	2014	I	Level 4: A cross-sectional sequential mixed-methods study on the usage and acceptance of insecticidal screens in the selected population.	• Indicates that there was a wide acceptance towards long-lasting insecticidal net screens.<br>• Mentions that reduction in both flies and cockroaches were significant upon usage of the long-lasting insecticidal net screens.
149	Poor Housing Quality Increases Risk of Rodent Infestation and Lassa Fever in Refugee Camps of Sierra Leone	Am J Trop Med Hyg	2007	J	Level 3b: A case-control study on the relationship between homes with a Lassa case and the location of rodent habitats.	• Indicates that one of the risk factors for Lassa infection is environment hygiene.<br>• Suggests that external hygiene – precisely the heightened opportunity for food and shelter for rodents – has a correlation with rodent burrows and risk of disease.
150	Evaluation of polyurethane resin injection for concrete leak repair	Case Stud Constr Mater	2019	I	Level 5: A study on the effectiveness of water leakage treatment with the use of injections of polyurethane resin.	• Mentions that the reduction in the permeability and subsequent water leakage is demonstrated upon usage of polyurethane resin injection. The injections can be used to fill cracks and reduce water leakage in various scenarios.
151	Domestic burglary drop and the security hypothesis	Crime Sci	2017	I	Level 2b: A cohort study on the relationship between security devices and burglary.	• Mentions that security devices for doors and windows enhances household safety, such as decreasing the risk of theft or burglary.
152	Are housing improvements an effective supplemental vector control strategy to reduce malaria transmission? A Systematic Review	School of Public Health, Georgia State University	2014	I	Level 2a: A systematic review to evaluate the relationship between household improvements and malaria transmission.	• Concludes that house improvements including screenings windows or closing eaves can reduce contact with malaria vectors.<br>• Indicates that not all housing improvements would present with equal efficacy due to housing differences.
153	Measures to control *Phlebotomus argentipes* and visceral leishmaniasis in India	J Arthropod Borne Dis	2016	I	Level 5: A review to propose measures to control two particular VBDs in India.	• Presents measures such as insecticide-treated bed nets, environmental modification and indoor residual spraying for vector control.<br>• Considers alternatives for measures such as environmental modification with recognition of the limitations posed by alternatives such as mud and lime mixtures.

154	Housing gaps, mosquitoes and public viewpoints: A mixed methods assessment of relationships between house characteristics, malaria vector biting risk and community perspectives in rural Tanzania	Malar J	2018	I	Level 4: A mixed method study to understand the relationship between household characteristics and malaria transmission, and knowledge, views and perceptions of community members regarding this relationship	• Mentions that less well-off populations cannot afford modern building materials for household modifications.<br>• Indicates that in the studied rural population, substantial proportions of individuals still reside in homes with open eaves, unscreened windows and doors with gaps.
155	Window screening, ceilings and closed eaves as sustainable ways to control malaria in Dar es Salaam, Tanzania	Malar J	2009	I	Level 4: A study with a cross-sectional household survey to estimate usage of screenings, ceilings and closed eaves as well as to evaluate expenditure and reasons behind installation.	• Reports that a significant number of respondents lacking the measure believed that it was too expensive.<br>• Concludes that further promotion is necessary for the increase of these highly acceptable practices.
156	Risk factors for Indian kala-azar	Am J Trop Med Hyg	2005	I	Level 3b: A case-control study to understand the various risk factors for kala-azar.	• Indicates that mud plastered walls are a potentially significant risk factor for the VBD kala-azar (visceral leishmaniasis).
